# Phenotypic analysis combined with tandem mass tags (TMT) labeling reveal the heterogeneity of strawberry stolon buds

**DOI:** 10.1186/s12870-019-2096-0

**Published:** 2019-11-19

**Authors:** Ling Guan, Mizhen Zhao, Yaming Qian, Hongmei Yu, Jin Xia, Ejiao Wu

**Affiliations:** Institute of Pomology, Jiangsu Academy of Agricultural Sciences Jiangsu Key Laboratory for Horticultural Crop Genetic improvement, Nanjing, 210014 China

**Keywords:** Strawberry, Stolon buds, Phenotypic observation, Differentially expressed proteins, Tandem mass tags

## Abstract

**Background:**

Ramet propagation in strawberry (*Fragaria* × *ananassa*) is the most effective way in production. However, the lack of systematically phenotypic observations and high-throughput methods limits our ability to analyze the key factors regulating the heterogeneity in strawberry stolon buds.

**Results:**

From observation, we found that the axillary bud located in the first node quickly stepped into dormancy (DSB), after several bract and leaf buds were differentiated. The stolon apical meristem (SAM) degenerated as the new ramet leaf buds (RLB), and the new active axillary stolon buds (ASB) differentiated continually after the differentiation of the first leaf. Using the tandem mass tags (TMT) labeling method, a total of 7271 strawberry proteins were identified. Between ASB and DSB, the spliceosome DEPs, such as Ser/Arg-rich (SR) and heterogeneous nuclear ribonucleoprotein particle (hnRNP), showed the highest enrichment and high PPI connectivity. This indicated that the differences in DEPs (e.g., SF-3A and PK) at the transcriptional level may be causing the differences between the physiological statuses of ASB and DSB. As expected, the photosynthetic pre-form RLB mainly differentiated from ASB and DSB judging by the DEP enrichment of photosynthesis. However, there are still other specialized features of DEPs between RLB and DSB and between ASB and DSB. The DEPs relative to DNA duplication [e.g., minichromosome maintenance protein (MCM 2, 3, 4, 7)], provide a strong hint of functional gene duplication leading the bud heterogeneity between RLB and DSB. In addition, the top fold change DEP of LSH 10-like might be involved in the degeneration of SAM into RLBs, based on its significant function in modulating the plant shoot initiation. As for RLB/ASB, the phenylpropanoid biosynthesis pathway probably regulates the ramet axillary bud specialization, and further promotes the differentiation of xylem when ASB develops into a new stolon [e.g., cinnamyl alcohol dehydrogenase 1 (CAD1) and phenylalanine ammonia-lyase 1 (PAL1)].

**Conclusions:**

By using phenotypic observation combined with proteomic networks with different types of strawberry stolon buds, the definite dormancy phase of DSB was identified, and the biological pathways and gene networks that might be responsible for heterogeneity among different stolon buds in strawberry were also revealed.

## Background

*Fragaria*, the strawberry, belongs to the rose family, and is an herbaceous perennial that can be easily asexual propagated by stolons or by crown division [1]. For this, *Fragaria* × *ananassa* Duch. as the cultivated strawberry, although originated ~ 250 years ago, could be widely and commercially produced by 76 countries around the world [2, 3]. Nowadays, both the cultivated (*F. ananassa*) and wild (*F. vesca*) strawberry genome were sequenced [4, 5]. They are widely preferred as model plant by horticultural researchers, especially those studying pomology. However, the cultivated *F*. × *ananassa* emerged from the hybridization of two wild octoploid species, both of which were descendants from the merger of four diploid progenitor species into a single nucleus [5]. It is highly heterozygous and causes high variability and genetic segregation in progeny seedlings. In stolon asexual propagations, the first node bud usually stays dormant, and only the buds of the second node have the ability to form ramets [3]. Thus, new insights into the mechanisms enabling stolon buds to produce either dormant or active buds to produce plantlets are crucial for improving strawberry productivity, and the establishment of the labor-saving cultivation methods.

Morphologically, a strawberry stolon is a special lateral branch of the crown, which originates from the mother plant’s axillary meristems with its subtending nodal ramet structure [6, 7]. Anatomically, a strawberry stolon consists of a large proportion of thick cortex and a relatively small proportion of phloem, xylem, and pith for transporting water, ions, and photoassimilates between the mother plant and ramets [8, 9]. The process of ramet formation by the second node of a strawberry stolon can be summarized as follows: the second node degenerates into the first leaf of a future ramet and is enclosed by its bracts. These degenerated apices will forming the compressed stems with short internodes that are called the crown [10]. The adventitious roots are formed from the bases of this newly formed compressed crown. Upon the completion of the rooting process, the lateral bud on the second node begins to elongate to form a new stolon. This newly formed stolon is not a continuous part of the mother plant, but rather a lateral bud located on the first plantlet’s axil. Next, the second or third ramets are sequentially formed under favorable environmental conditions [3].

In phenology, the axillary buds located on the leaves of a compressed crown usually form stolons under long-day, warm-temperature conditions [3], or after long periods of chilling [11, 12]. However, branch crown formation usually occurs under conditions wherein plants produce few stolons, such as under the short photoperiods [13]. Previous research has demonstrated that the apices on the actively developing branch crowns have an inhibitory effect on the growth of undeveloped axillary crown buds (UACBs), and this apices’ apical dominance can even result in the other UACBs remaining dormant until the apices are removed, other UACB’s dormancy will be released [10, 14]. However, the mechanism by which the first node buds of cultivated strawberry stolons usually remain in dormancy is still unknown today, and even when this dormancy is released under favorable conditions, the first node buds mostly have no ability to form ramets; how they develop into new stolon branches is, thus, not clear. In addition, the regulation of the mechanism underlying the dormancy of a stolon branch bud in the first node and the activity of the newly formed ramet located in the second node, are not clearly understood.

Previous studies on strawberry stolon have mostly focused on the mechanism of its formation or on the dictates of the flowering-runnering decision. According to a recent study, the DELLA protein seems to be an important factor controlling runner formation during asexual reproduction in strawberry [15]. Gibberellic acid (GA) biosynthesis in the axillary meristem is essential for inducing stolon differentiation. The possibility of the *FveGA20ox4* gene regulating the flowering-runnering decision in strawberry has been revealed previously [7]. Studies on the differences between the first and second nodes of strawberry stolon have always focused on the internode differences instead of the differences between buds. For example, the two-dimensional gel electrophoresis was used for comparing the proteomic profiles of the strawberry stolon internodes I-1 and I-2 [16]. They found that the ubiquitin-proteasome and sugar-hormone pathways might be important during adventitious root formation at the second node of new clonal plants. Results of quantifying the movement of resources and their allocation between mother plants and daughter ramets along *Fragaria* stolons with respect to hierarchy, the results showed that the stolon anatomy develops rapidly at the apical end, facilitating hierarchical ramet development, which is evident as a basipetal increase in hydraulic conductivities. The rapid development of transport tissue functionality enables young unrooted ramets to acquire water as well as mineral ions disproportionally with respect to older ramets, in order to supply an expanding leaf area [9].

However, there has been no systematic study on the heterogeneity of all three types of cultivated strawberry stolon buds. Thus, based on the inspiration of previous studies, we first elucidate the developmental characterizations of the first and second node buds at different developmental stages through anatomical observation. Subsequently, we illustrate the definite dormancy phase of the bud of the first node and the detailed developmental processes of ramet formation and ramet axillary bud elongation in the second node of strawberry stolon using stereomicroscopy and scanning electron microscopy. Considering the proteomics utilization, especially the tremendous advantages in method of TMT in digging the DEPs among multiple groups of plant materials [17–21]. Lastly, we lay a foundation for understanding the mechanisms of strawberry stolon phenotype and bud development at the protein level by using TMT labeling method. Through phenotypic observation combined with proteomic networks analysis of different types of strawberry stolon buds, the definite dormancy phase of DSB is identified, and the biological pathways and gene networks that might be responsible for heterogeneity among different stolon buds in strawberry are also revealed.

## Results

### Stolon anatomy

**Fig. 1:**
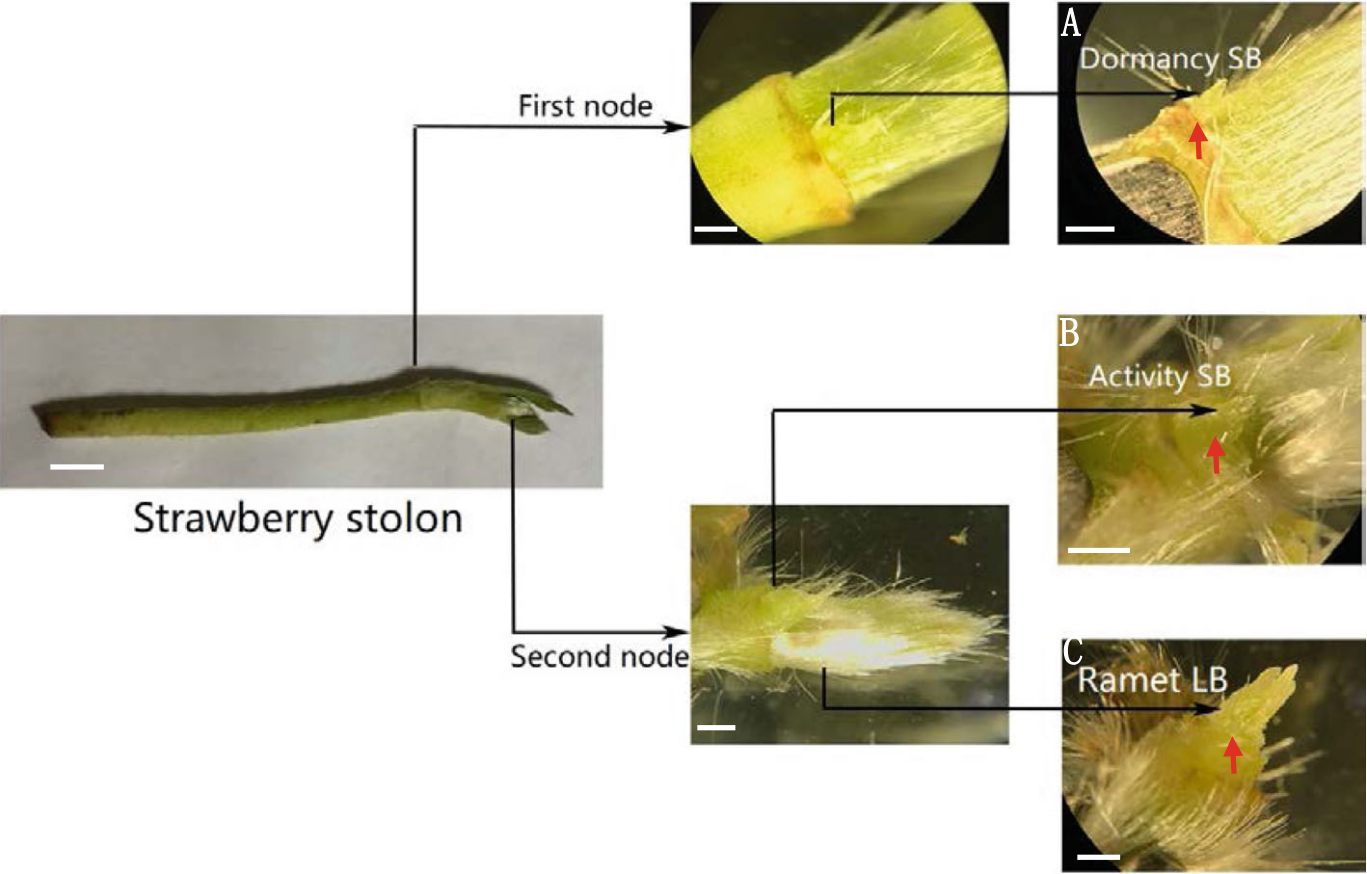
Three bud physiological statuses in the first node (**a**) dormancy shoot bud (DSB) and the second node (**b**) activity shoot bud (ASB) and (**c**) ramet leave bud (RLB)] of strawberry stolons. The scale bar in the images showing the whole strawberry stolon is 1 cm, and those in the other images are 1 mm

**Fig. 2:**
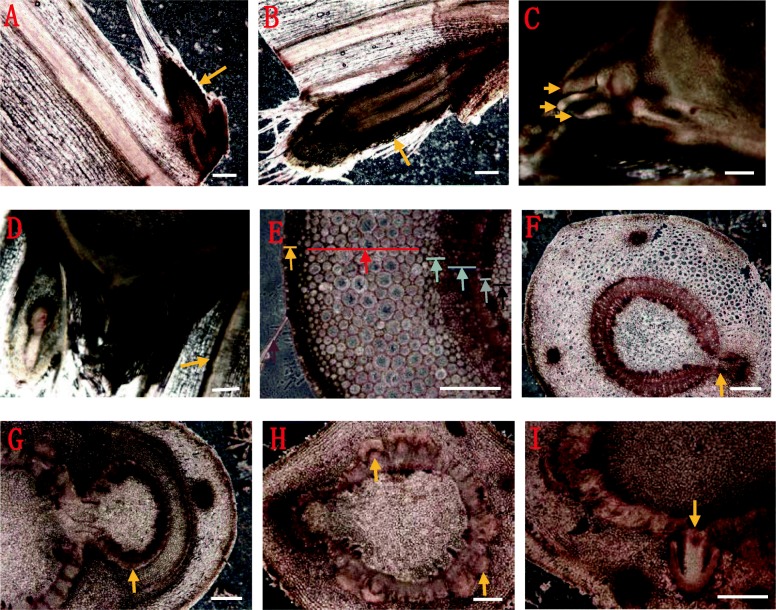
Slice observation of strawberry stolon. (**a**) Growth arrest in the first node and its stepping into dormancy; (**b**) Continuous development as a new stolon branch upon dormancy release; (**c**) Arrows indicate the trifoliolate ramet leave buds in the second node; (**d**) Vascular bundles (arrow) of the new developing stolon in the second node connected to the primary stolon; (**e**) The tissues from the outside to the inside of the stolon are epidermal hair (purple) and epidermis (orange), thick cortex (red), cambium (green; composed of a large number of parenchymatous cells), phloem (blue), xylem (grey), and pith (black); (**f–g**) vascular tissue of the lateral buds on the first (F, orange arrow) and second nodes (G, orange arrow) of the strawberry stolon connected inward with the vascular tissue of the primary stolon; (**h-i**) adventitious root primordia, which originating from the cambium. The scale bar is 0.5 mm in images (**a-b**) and (**f-i**) 1 mm in images (**c-d**) and 0.3 mm in image (**e**)

Anatomical observation showed that the first node of the stolon (10 cm length) in cultivated strawberry (*F*. × *ananassa* Duch.) was extremely small and could be easily missed. When the bract of the first node was peeled off, a tiny bud (Fig. 1a) was observed. In contrast, upon observation of the second node, two different types of buds were observed underneath the bract—one was a plump bud (Fig. 1b), and the other was a leaf cluster mixed with several developing leaf buds (Fig. 1c). Slice observation showed that the buds in the first node stopped growing and stepped into dormancy at an early stage (Fig. 2a). The dormancy of the bud located at the first node could be released only under favorable environmental conditions, and it continued to develop into a new stolon branch (Fig. 2b). The leaf buds, located inside of the bract of the second node, showed a distinct trifoliolate structure (Fig. 2c), and the vascular bundles of the newly formed stolon, which is laterally located on the leaf buds, were connected inward with the primary stolon (Fig. 2d). The structure of the strawberry stolon was observed by cross-sectional anatomy. The tissues, from outside to inside, of the stolon included the epidermal hair and epidermis, thick cortex, cambium, phloem, xylem, and the pith, which are composed of a large number of parenchymatous cells (Fig. 2e). These two lateral buds on the first and second nodes of stolon were inwardly connected with the primary stolon in a similar pattern (Fig. 2f-g). At the base of the second node, which is connected to the terminal strawberry stolon buds, there are numerous adventitious root primordia (Fig. 2h-i). Each adventitious root primordium originated from the cambium tissue, which consists of meristematic cells containing dense cytoplasm and swollen nuclei (Fig. 2e, i).

### Stereoscopic and SEM observation of developing stolon buds

#### Dormancy bud in the first node

**Fig. 3:**
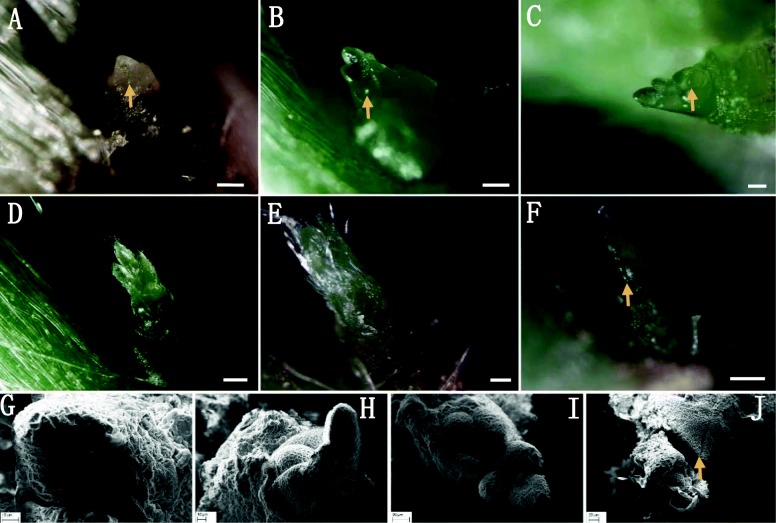
Dormancy shoot bud (DSB) developmental phases in the first node. **a–c** Three typical developmental phases of DSB (arrow) at the early stage of primary stolon elongation. **d–e** DSB covered by a young trifoliolate leaf bract. **f** Closed bract with a DSB inside. **g–j** Scanning electron microscope (SEM) photomicrographs of DSB in (**a–c**) and (**f**). The scale bar is 10 μm in (**a**) and (**g–h**) 25 μm in B, 50 μm in (**c**), 100 μm in (**d–e**) 150 μm in F, and 20 μm in (**i–j**)

In order to acquire more details on the developmental characteristics of the first and the second node buds of the strawberry stolon, stolon buds in different developmental phases were observed under the stereomicroscope and scanning electron microscope (SEM), respectively. In the early stage of stolon elongation (when the stolon length was 4–10 cm), the buds on the first node of the stolon grew with the subsequent development of the stolon at the early stage (Figs. 3a–c). For example, when the stolon was 4 cm in length, a tiny growing point located in the center of a trifoliolate bud could be seen after the outermost bract was peeled off from the first node (Fig. 3a). Continually, along with the growth of stolon (when the stolon length was 6–7 cm), the top trifoliolate leaf bud on the first node developed further, and the growth point at the central base of the buds also grew (Fig. 3b–c). With the further elongation of the primary stolon, when the primary stolon reached a length of 8–9 cm, the original trifoliolate bud gradually developed into a young trifoliolate bract and was densely covered with trichomes (Fig. 3d–e). When this young trifoliolate was peeled off sequentially, another tightly closed thin trifoliolate bud could be seen protecting the underlying growing point (Fig. 3f). This is a landmark at which the bud of the first node in the strawberry stolon ceases to develop and enters into dormancy; under continual observation, when the primary stolon elongated further, this thin trifoliolate bud structure showed no change. Our conclusion was further confirmed under the magnified observation of SEM; the first trifoliolate bud under the bract of the first node continuously develops into a young trifoliate, with the development of the primary stolon occuring at an early stage of stolon development or elongation (Figs. 3g, j). Similarly, when this young trifoliolate leaf was peeled off, the structure of the thin, tightly closed trifoliolate bract was visible (Fig. 3j). With all this, the outermost new trifoliolate leaf bud and the inner growing point ceased to develop, and showed no further development while the primary stolon elongated continually, indicating its stepping into a state of dormancy.

#### Activity shoot bud in the second node

**Fig. 4:**
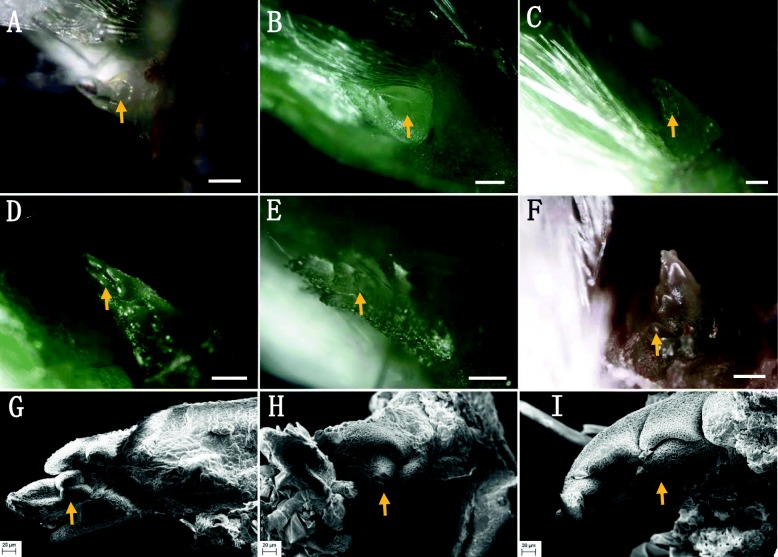
Activity shoot bud (ASB) development in the second node. **a–b** ASB primordium located at the center of the developing and matured bract. **c–e** ASB apex development when the matured bract was peeled off. **f** ASB structure when the young apex of ASB was peeled off again and a new growth point (arrow). **g–i** are the respective SEM images for (**d–f**); in these images, the trifoliolate apex can be seen to exhibited a high developmental activity. The scale bar is 50 μm in (**a-d**) 100 μm in (**e–f**), and 20 μm in (**g–i**)

The active stolon bud (ASB) under the outermost bract of the second node of a strawberry stolon is quite different from the DSB located in the first node, showing an active development state throughout the whole process of primary stolon development (Fig. 4). The process of ASB developmental inside the second node also showed that the trifoliolate leaf bud developed first (Figs. 4a–d). When this trifoliolate leaf bud developed into a young trifoliolate (Fig. 4e), the new inner trifoliolate could be seen (Fig. 4f) and, at the same time, a new growth point started to develop near the trifoliolate cluster (Fig. 4f). SEM observation revealed further details of the development of trifoliolate leaf buds, which exhibited a high developmental activity (Figs. 4g–i).

#### Ramet buds in the second node

**Fig. 5:**
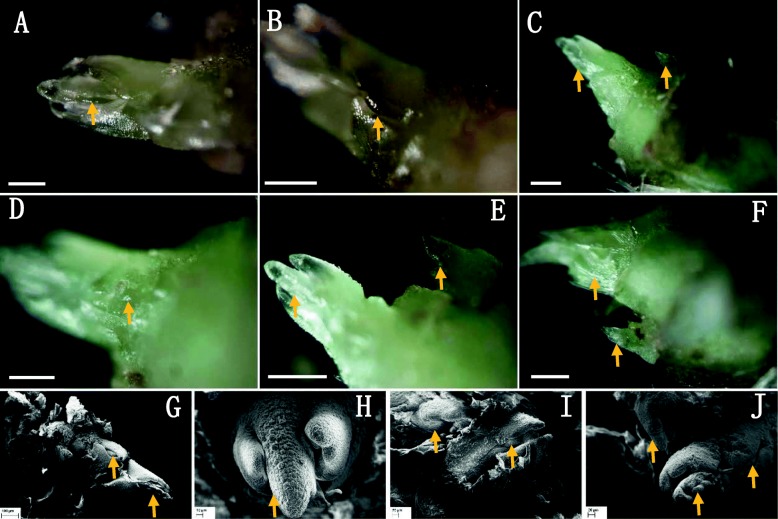
Ramet leave bud (RLB) development in the second node of a strawberry stolon. **a** Pure trifoliolate leave enclosed in the bract. **b** Newly formed bract, with a new primordium of trifoliolate leaves inside. **c**–**e** Development of ramet leave buds and laterally located newly formed stolon buds. **f** Young trifoliolate cluster and laterally located new stolon in the second node. **g** and **h** Detailed exhibition of ramet leave buds cluster and the bird's eye perspective of a single developing leaf bud, respectively. **i**–**j** Development of laterally located newly formed stolon buds with the growth of apical ramet buds. The scale bar is 150 μm in (**a–b**) and (**d–e**), 300 μm in (**c** and **f**) 100 μm in *G*, 10 μm in (**h**) and 20 μm in (**i–j**)

Unlike the axillary development of DSBs and ASBs, the developmental process of the strawberry ramet leaf bud (RLB) is relatively simple and rapid (Fig. 5). The apical region of a strawberry stolon contains multiple leaf bud primordia; when one leaf primordium gradually develops into a young trifoliolate, the next leaf primordium is initiated out as a visible developing trifoliolate (Figs. 5a, b). Subsequently, each leaf primordium develops into a young leaf, orderly, to form a young leaf cluster (Fig. 5c–f). The stolon branch developing activity is located laterally to these leaf clusters (Figs. 5e, f). SEM observation showed that the trifoliolate bract first to grow out is located at the top of each trifoliolate primordium to protect the inner part (Fig. 5g). Each trifoliolate bract was tightly connected with the others in a complementary manner (Fig. 5g) to save the limited growth space. As a result, each connected young trifoliolate in the ramet of the second node is well positioned, and the active stolon bud located adjacent to them (Figs. 5i, j).

### Protein profiles among different strawberry stolon buds

For searching the regulatory factors involved in modulating the heterogeneity of strawberry stolon buds at the proteomic level, DEPs among different types of buds were assessed by profiling the proteome using the TMT labeling system. The expression profiles of the proteins extracted from DSBs (labeled with 126, 127 N, 127C), ASBs (labeled with 128 N, 128C, 129 N), and RLBs (labeled with 129C, 130 N,130C) on a 10-cm long strawberry stolon were analyzed.

#### LC-MS/MS

**Fig. 6:**
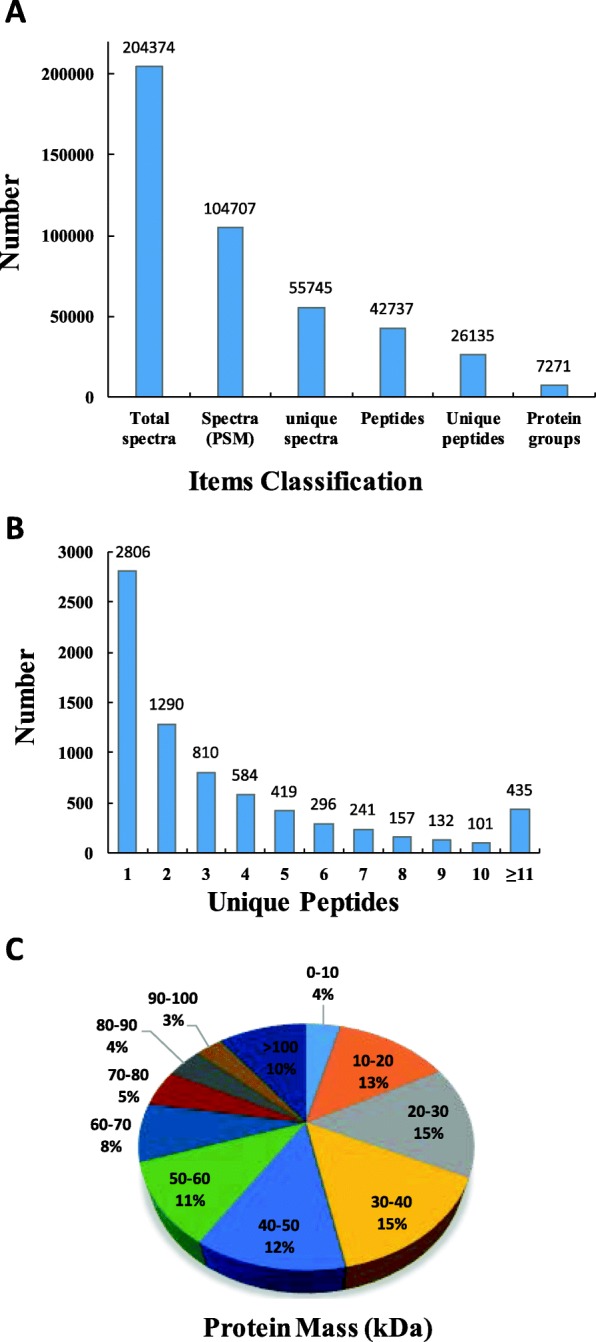
Results of the tandem mass tags (TMT)–based liquid chromatography-tandem mass spectrometry (LC-MS/MS) identification of the stolon buds of the Ning Yu variety of strawberry. **a** Classification of the items used for identifying proteins; **b** Number of unique peptides that were matched to each identified protein. **c** Distribution of the average molecular masses of identified proteins

A total of 42,737 peptide fragments, of which 26,135 were unique peptides corresponding to a total of 7271 proteins (Fig. 6a), were assessed by TMT-based LC-MS/MS mass spectrometry identification and a search against the P20180400239_hebing_76764 database (detailed information in the Materials and Mathods) employing MASCOT engine integrated with Proteome Discoverer 1.4 software (Additional file 1: Figure S1, Additional file 2: Figure S2). A 1.2-fold-change cut-off with *P*-value<0.05 was used to indicate significant changes in the abundance of DEPs among different strawberry stolon buds (Additional file 2: Figure S2). By analyzing the quality control data, we found that the TMT results that were achieved by using a high-quality Q Exactive mass spectrometer was reliable. The accuracy and high resolution achieved in our experiment can maintain good quality deviation during the process of data acquisition, and produce high-quality MS1 and MS2 spectrograms. The quality deviation of all identified peptides was mainly within 10 ppm (Additional file 3: Figure S3), indicating that the identification results were accurate and reliable. When the rigid analyzing tool of MASCOT (FDR < 0.01) was used for judging each MS2 spectrogram, we obtained an ideal score with a median of 34.06, and more than 86.21% peptides scored higher than 20 (Additional file 4: Figure S4). The protein ratio (approximately 1.0) distribution of the three groups (ASB/DSB, RLB/DSB, and RLB/ASB) are shown in Additional file 5: Figure S5.

#### Features of identified proteins

The distribution of unique peptides defining each protein is shown in Fig. 6b; over 61% of proteins, included at least two unique peptides (Additional files 10 and 11: Table S1-Table S2). The average molecular mass of the identified gene products ranged from 10 to 70 kDa (Fig. 6c). The t (PI) distribution of the identified proteins was mainly in the range of 5.0–10.0, with most PIs ranging from 6.0 to 7.0 (Additional file 6: Figure S6). Comparisons between the DSB, ASB, and RLB groups led to the identification of 1307 ASB/DSB (including 691 up-regulated and 616 down-regulated), 363 RLB/DSB (168 up-regulated and 195 down- regulated) and 626 RLB/ASB (256 up-regulated and 370 down-regulated) DEPs (Additional file 12: Table S3). The variation among the three biological replicates of each group (DSB, ASB, and RLB) was calculated according to their quantitative data, with most proteins exhibiting less than 20% variation among replicates Additional file 7: Figure S7), indicating the high quality and repeatability of the data.

For exhibiting the DEPs among each group in detail, we identified top 10 up-regulated and top 10 down-regulated DEPs according to their fold changes (Additional file 12: Table S3) [in total of 1307 in ASB/DSB, 691 up-, 616 down-; 363 in RLB/DSB, 168 up- and 195 down-; and 626 in RLB/ASB, 256 up- and 370 down-regulated DEPs]. These top DEPs were selected out and listed in Table 1, and by further analyzing, there could be found 5, 1 and 6 common top DEPs among (ASB/DSB and RLB/DSB), (RLB/DSB and RLB/ASB) and (ASB/DSB and RLB/ASB), respectively, and additionally with 3 common top DEPs were found in all above three groups (Additional file 13: Table S4). All these 5 common top DEPs between the groups of ASB/DSB and RLB/DSB were down-regulated DEPs, they were [cucumisin-like], [serine carboxypeptidase-like 27], [ornithine decarboxylase-like], [36.4 kDa proline-rich protein], and [ribulose bisphosphate carboxylase small chain, chloroplastic-like protein]. Indicating that all these 5 common top DEPs are highly expressed in DSB when compared with ASB and RLB, and we could speculate that they are mainly involved in stimulating the developmental phases or promoting the dormancy process of the first node of DSB in a strawberry stolon. However, between the groups of RLB/DSB and RLB/ASB, only 1 up-regulated common top DEP of [pentatricopeptide repeat-containing protein] was found.

**Table 1:** Top 10 of up-regulated and down-regulated differentially expressed proteins between groups

Groups	No.	Protein ID	Description	Coverage	Fold change	*P*-value
ASB/DSB	up-1	XP_004304418.1	fasciclin-like arabinogalactan protein 12	20.73	6.23	0.00186255
	up-2	FANhyb_rscf00000173.1.g00014.1	hypothetical protein CARUB_v10021660mg	22.06	5.29	0.02442000
	up-3	FANhyb_rscf00000386.1.g00003.1	fasciclin-like arabinogalactan protein 12	20.73	4.78	1.3548E-07
	up-4	XP_011462288.1	glycine-rich cell wall structural protein	50.36	4.64	0.015453
	up-5	XP_004299681.1	blue copper protein-like	14.21	4.52	0.004586
	up-6	FANhyb_rscf00004026.1.g00001.1	glucuronoxylan 4-O-methyltransferase 3-like	7.360	3.80	0.00520136
	up-7	FANhyb_icon00011962_a.1.g00001.1	fructokinase-5	38.28	3.61	0.00072744
	up-8	XP_011458504.1	anthocyanidin 3-O-glucosyltransferase 7-like	19.91	3.53	0.00100593
	up-9	FANhyb_rscf00000027.1.g00021.1	zinc finger protein	2.580	3.47	0.00693375
	up-10	FANhyb_rscf00000295.1.g00005.1	histone H2A.1	37.84	3.43	0.00117361
	down-1	XP_004307317.1	cucumisin-like	7.830	0.34	0.01617526
	down-2	XP_004307401.1	serine carboxypeptidase-like 27	11.79	0.35	0.01705444
	down-3	FANhyb_rscf00000020.1.g00007.1	pectinesterase	4.170	0.37	0.03259767
	down-4	XP_004297134.2	ornithine decarboxylase-like	4.660	0.38	0.00237870
	down-5	FANhyb_rscf00000011.1.g00007.1	36.4 kDa proline-rich protein	9.320	0.38	0.00620498
	down-6	FANhyb_icon00014184_a.1.g00001.1	putative laccase-9	2.760	0.41	0.01597461
	down-7	XP_004303137.1	ribulose bisphosphate carboxylase small chain, chloroplastic-like	31.87	0.42	0.01480696
	down-8	FANhyb_rscf00001104.1.g00003.1	aquaporin TIP2-1	7.260	0.43	0.00599268
	down-9	FANhyb_icon00031506_a.1.g00001.1	putative 4-hydroxy-4-methyl-2-oxoglutarate aldolase 2	47.83	0.45	0.00430290
	down-10	XP_004293363.1	fruit protein pKIWI501-like	50.00	0.45	0.02295962
RLB/DSB	up-1	XP_004303183.1	protein LIGHT-DEPENDENT SHORT HYPOCOTYLS 10-like	13.88	3.24	0.01882948
	up-2	FANhyb_rscf00005475.1.g00002.1	pentatricopeptide repeat-containing protein At4g38150-like	10.04	2.29	0.01396803
	up-3	XP_004307632.1	flocculation protein FLO11 isoform X3	2.470	1.76	0.00227661
	up-4	FANhyb_rscf00000173.1.g00014.1	hypothetical protein CARUB_v10021660mg	22.06	1.76	0.00017183
	up-5	FANhyb_icon00004730_a.1.g00001.1	squamosa promoter-binding-like protein 9	7.090	1.69	0.00267638
	up-6	FANhyb_rscf00000264.1.g00010.1	uncharacterized protein LOC101290827	12.68	1.61	0.03225374
	up-7	XP_011462288.1	glycine-rich cell wall structural protein	50.36	1.58	0.00426962
	up-8	XP_004299681.1	blue copper protein-like	14.21	1.58	0.00418736
	up-9	XP_004310149.1	mediator-associated protein 2	10.27	1.57	0.04857720
	up-10	FANhyb_rscf00005241.1.g00001.1	zinc-finger homeodomain protein 6	16.35	1.56	0.02325629
	down-1	XP_004307317.1	cucumisin-like	7.830	0.41	0.02484890
	down-2	XP_004297134.2	ornithine decarboxylase-like	4.660	0.41	0.00581982
	down-3	XP_004307401.1	serine carboxypeptidase-like 27	11.79	0.41	0.02026585
	down-4	FANhyb_rscf00001190.1.g00001.1	telomere repeat-binding factor 1-like	9.040	0.50	0.00752576
	down-5	FANhyb_rscf00000011.1.g00007.1	36.4 kDa proline-rich protein	9.320	0.51	0.01674637
	down-6	FANhyb_rscf00000159.1.g00006.1	glyceraldehyde-3-phosphate dehydrogenase B, chloroplastic	14.16	0.54	0.01030551
	down-7	FANhyb_rscf00005433.1.g00001.1	fasciclin-like arabinogalactan protein 13	8.540	0.55	0.00275099
	down-8	XP_004303137.1	ribulose bisphosphate carboxylase small chain, chloroplastic-like	31.87	0.56	0.03678190
	down-9	FANhyb_icon00037348_a.1.g00001.1	ribulose bisphosphate carboxylase small chain, chloroplastic-like	54.00	0.56	0.03512944
	down-10	XP_004297630.1	carbonic anhydrase 2 isoform X2	19.37	0.57	0.00032382
RLB/ASB	up-1	FANhyb_rscf00000329.1.g00005.1	1-aminocyclopropane-1-carboxylate oxidase 1-like	16.07	2.16	0.00162178
	up-2	FANhyb_rscf00001394.1.g00002.1	glutamate dehydrogenase 1	20.87	2.05	0.04362181
	up-3	FANhyb_icon00031506_a.1.g00001.1	putative 4-hydroxy-4-methyl-2-oxoglutarate aldolase 2	47.83	2.05	0.00751062
	up-4	XP_004293441.1	major latex allergen Hev b 5-like	68.04	2.04	0.00379106
	up-5	XP_004293363.1	fruit protein pKIWI501-like	50.00	2.00	0.00846049
	up-6	FANhyb_rscf00001104.1.g00003.1	aquaporin TIP2-1	7.260	1.98	0.00734669
	up-7	FANhyb_rscf00000704.1.g00001.1	putative formamidase C869.04 isoform X1	3.690	1.98	0.01632577
	up-8	FANhyb_rscf00005475.1.g00002.1	pentatricopeptide repeat-containing protein At4g38150-like	10.04	1.89	0.02607894
	up-9	FANhyb_rscf00000980.1.g00003.1	glutathione S-transferase F13-like	43.45	1.81	0.00588200
	up-10	XP_004291285.2	probable sarcosine oxidase	8.690	1.80	0.00202212
	down-1	XP_004304418.1	fasciclin-like arabinogalactan protein 12	20.73	0.23	0.00070797
	down-2	FANhyb_rscf00000386.1.g00003.1	fasciclin-like arabinogalactan protein 12	20.73	0.25	0.00001714
	down-3	FANhyb_rscf00004026.1.g00001.1	glucuronoxylan 4-O-methyltransferase 3-like	7.360	0.29	0.00706772
	down-4	XP_011457444.1	F-box protein SKIP14	2.380	0.30	0.00827485
	down-5	FANhyb_icon00004976_a.1.g00001.1	cytochrome P450 84A1	28.68	0.31	7.5325E-07
	down-6	XP_004294339.1	probable galacturonosyltransferase 12	3.000	0.31	0.01158193
	down-7	FANhyb_rscf00000173.1.g00014.1	hypothetical protein CARUB_v10021660mg	22.06	0.33	0.04426256
	down-8	XP_011462288.1	glycine-rich cell wall structural protein	50.36	0.34	0.02679911
	down-9	XP_004299681.1	blue copper protein-like	14.21	0.35	0.03378082
	down-10	FANhyb_rscf00002637.1.g00001.1	L-type lectin-domain containing receptor kinase IX.1-like	2.040	0.35	0.03378082
	down-10	FANhyb_rscf00002637.1.g00001.1	L-type lectin-domain-containing receptor kinase IX.1-like	2.04	0.35	0.03378082

Between ASB/DSB and RLB/ASB, all 6 common top DEPs showed consistent up-regulated expression in ASB/DSB and down-regulated expression in RLB/ASB. They were [fasciclin-like arabinogalactan protein 12] (double), [glucuronoxylan 4-O-methyltransferase 3-like], [aquaporin TIP2–1], [putative 4-hydroxy-4-methyl-2-oxoglutarate aldolase 2], and [fruit protein pKIWI501-like]. Thus, all these six common DEPs were highly expressed in ASBs, when compared with DSBs and RLBs, suggesting they are highly involved in the ASB developmental process. At last, these 3 common top DEPs in all three groups were [hypothetical protein CARUB_v10021660mg], [blue copper protein-like] and [glycine-rich cell wall structural protein], and also showed the consistent expression modes of ASB/DSB-up, RLB/DSB-up, and RLB/ASB-down. This implied that these 3 common top DEPs have the ability to promote the entry of DSBs into dormancy and to stimulate the development of ASBs at the same time. The other DEPs among the top 10 up- or down-regulated DEPs are specifically expressed in each group (Additional file 14: Table S5).

**Fig. 7:**
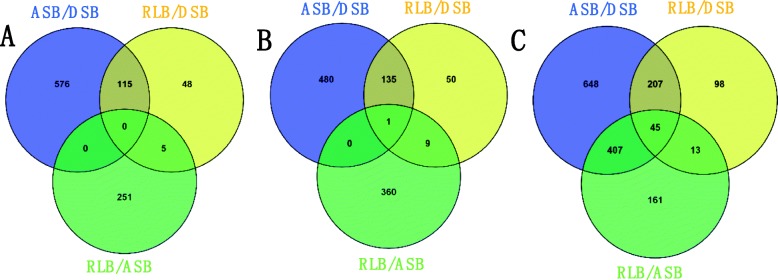
Venn diagram of co-up regulation (**a**) and co-down regulation (**b**), as well as total co-up plus co-down regulation (**c**) DEPs from each experimental group comparison

Additional large-scale analysis of DEPs between groups showing co-up regulation and co-down regulation was also carried out, as shown in the Venn diagram in Fig. 7 (detailed information in Additional file 15: Table S6, Additional file 16: Table S7, Additional file 17: Table S8). We found that no DEP showed co-up regulation; only one DEP of GDSL esterase/lipase showed co-down regulation among all three groups (Fig. 7a, b). When the total number of co-up regulated and co-down regulated proteins was counted, 45 DEPs common to all three groups were found (Fig. 7c). Among all statistics, one group of data showed special performance, that is, ASB/DSB and RLB/ASB have almost no co-up or co-down proteins (Fig. 7a, b), separately, but when we calculated the total co-up and co-down DEPs, 407 common DEPs could be found between groups of ASB/DSB and RLB/ASB, simultaneously (Fig. 7c). These results indicated that each of the 407 DEPs shared common down-regulated or up-regulated expression patterns in the groups of DSB and RLB when compared with ASB. This suggests that all these proteins have dual functions in stimulating the RLB formation while promoting the DSBs stepping into dormancy; however, they also have an antagonistic effect on the ASB development in strawberry stolons, simultaneously. Thus, exploring the regulatory mechanisms of these 407 DEPs is of great significance to clarify the dormancy of first node DSBs and the formation of the second node RLBs.

### Bioinformatics analysis

All DEPs detected by MS were subjected to a bioinformatics analysis for further classification.

#### Cluster analysis

**Fig. 8:**
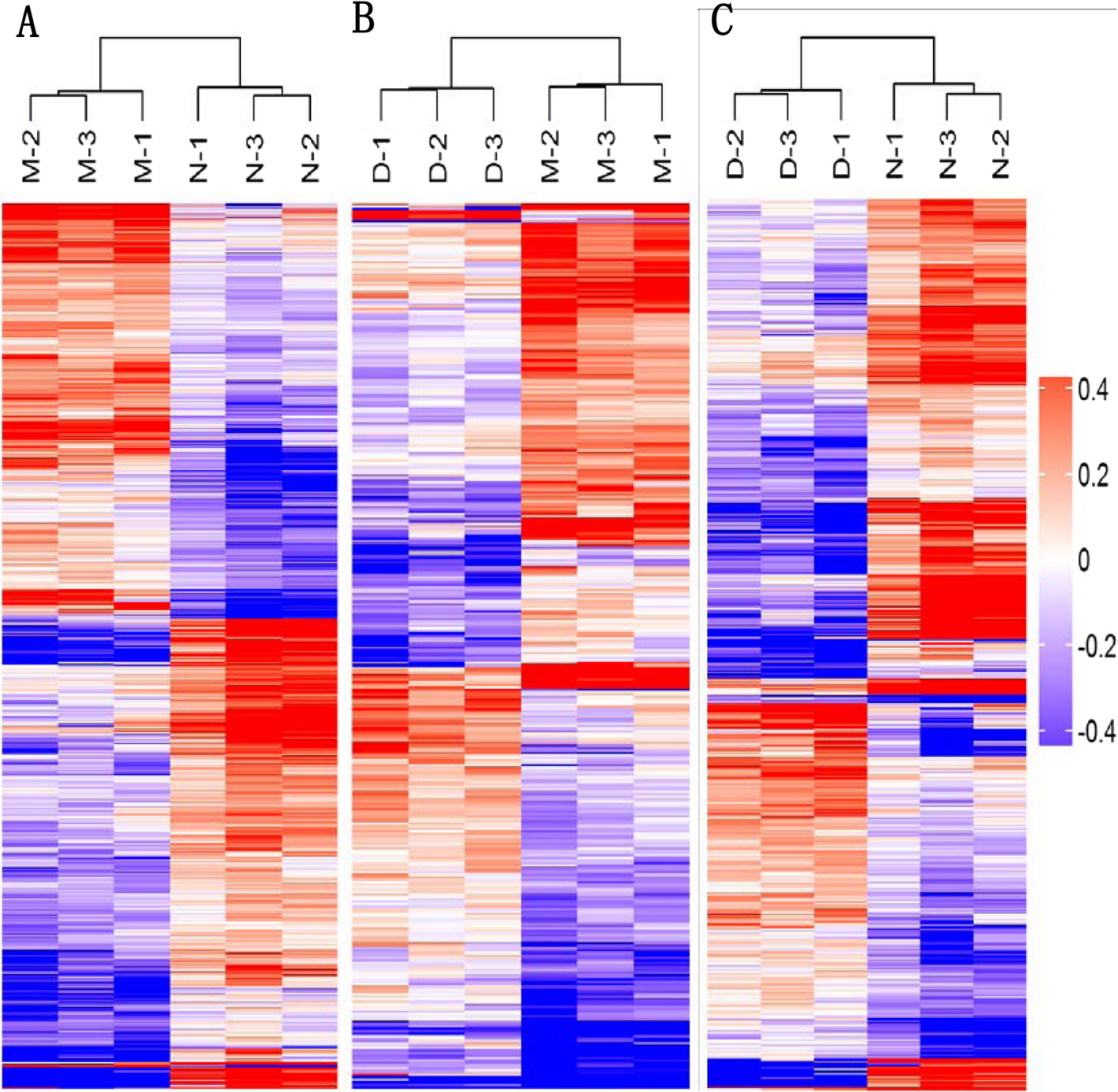
Cluster analysis of differentially expressed proteins. Through horizontal comparison, samples could be classified into three categories, suggesting that the selected DEPs could effectively distinguish between samples. A vertical comparison indicated that proteins could be classified into two categories with opposite directional variation, demonstrating the rationality of the selected DEPs. M, N, and D represent the DSB, ASB, and RLB groups, respectively. **a** is DSB/ASB, (**b**) is RLB/DSB, and (**c**) is RLB/ASB

The hierarchical clustering results were expressed as a respective heat map (Fig. 8). By a horizontal comparison, the samples could be classified into three categories: DSB, ASB, and RLB. Suggesting that the selected DEPs could be effectively distinguished between samples with high accuracy. Through a vertical comparison, the selected proteins could be classified into two categories with opposite directional variation, which showed the expression patterns of DEPs in three groups (Additional file 8: Figure S8), demonstrating the rationality of the selected DEPs. The cluster analysis, thus, supported that the DEPs screened through our experiment were accurate.

#### GO functional annotation and analysis

**Fig. 9:**
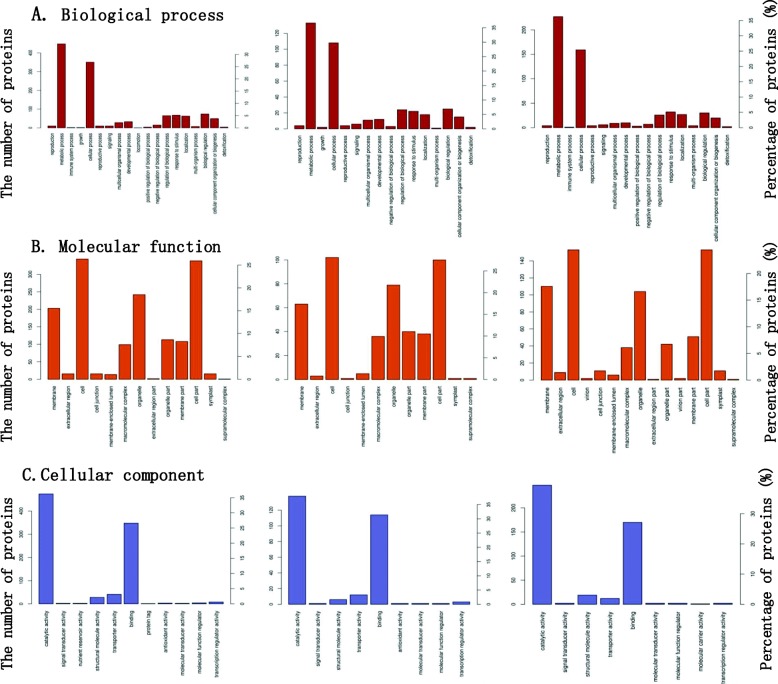
Gene ontology annotation of differentially expressed proteins (DEPs) among groups. The X-axis represents the Gene ontology functional classification. The main Y-axis represents the number of DEPs, and the secondary Y-axis represents the classified DEP ratio in the respective group’s total DEPs (ASB/DSB, RLB/DSB and RLB/ASB). **a** Biological process. **b** Molecular function. **c** Cellular component

The DEPs (1307, 363, and 626) between ASB and DSB, RLB and DSB, and RLB and ASB groups corresponded to 1931, 1194, and 1276 functional annotations, respectively (Additional file 18: Table S9, Additional file 19: Table S10, Additional file 20: Table S11). The DEPs were individually analyzed against the Gene Ontology (GO) database using three sets of ontologies: biological process, molecular function, and cellular component (Fig. 9). The analysis showed that among ASB/DSB, RLB/DSB, and RLB/ASB, numerous DEPs could be classified in the same GO category (Additional file 18: Table S9, Additional file 19: Table S10, Additional file 20: Table S11). The top two common *biological process* categories were metabolic process (over 35%) and cellular process (over 25%). The top four common *molecular function* categories were catalytic activity (over 35%), binding (over 25%), transporter activity, and structural molecule activity. The top four common *cellular component* categories were cell (over 25%), cell part (over 25%), organelle (over 15%), and membrane proteins (over 15%). A small number of other DEPs existed in cellular component categories, including membrane part, organelle part, and macromolecular complex, with the ratio of approximately 10%. For further exhibition of the top 20 enriched GO terms (Additional file 9: Figure S9), we know detailed infirmation of functional proteins in *biological process* (*BP*) of ASB/DSB were oxidation-reduction process (~ 100 DEPs) and regulation of RNA metabolic process (Additional file 9: Figure S9A), in RLB/DSB, they were DNA metabolic process and photosynthesis, with the same number of DEPs (14), as well as DNA conformation change and replication (Additional file 9: Figure S9B), whereas for RLB/ASB (Additional file 9: Figure S9C), there were small numbers of DEPs, which were classified into the secondary metabolic process, one-carbon metabolic process, secondary metabolite biosynthetic process, and phenylpropanoid metabolic process.

*Molecular function (MF)* analysis showed that the GO terms in ASB/DSB were catalytic activity (~ 450 DEPs) and oxidoreductase activity (over 100 DEPs), and DNA binding (Additional file 9: Figure S9A). In RLB/DSB, the MF proteins were DNA binding and helicase activity (Additional file 9: Figure S9B). In the RLB/ASB MF GO terms, a large numbers of DEPs belonged to catalytic activity (~250), oxidoreductase activity (~ 60), and protein dimerization activity (Additional file 9: Figure S9C).

*The cellular component (CC)* terms in ASB/DSB were thylakoid and thylakoid part and chromatin. Combining the GO terms identified in the MF and BP analysis above, we found that the differences existed mainly at the RNA level (Additional file 9: Figure S9A). Between the groups of RLB and DSB, the GO terms were thylakoid (22), thylakoid part (16), plastid thylakoid (14), chloroplast thylakoid (14), photosynthetic membrane (14), and thylakoid membrane (12) (Additional file 9: Figure S9B). This suggested that the differences between RLB and DSB mainly occurred in terms of their capacity for photosynthesis. In RLB/ASB, all DEPs of CC were functional compartments of chromosome- or DNA-related proteins, and showed a low coincidence trend (Additional file 9: Figure S9C).

#### KEGG pathway analysis

**Fig. 10:**
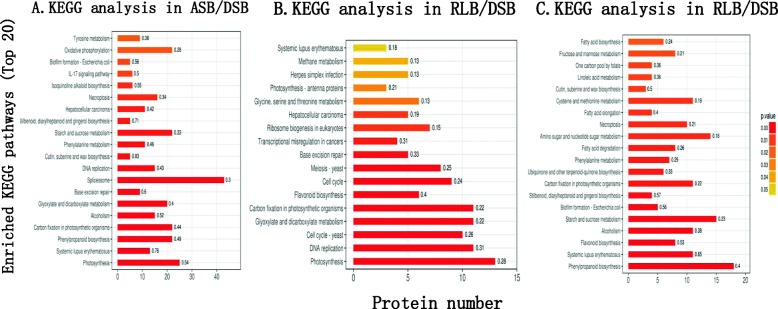
KEGG pathway analysis between different groups of ASB/DSB (**a**), RLB/DSB (**b**), and RLB/ASB (**c**)

By searching the major biological pathways and relevant regulatory processes involved in the Kyoto Encyclopedia of Genes and Genomes (KEGG), we analyzed all DEPs among groups (Fig. 10). The results indicated that the spliceosome (43 DEPs, as shown below) and ribosome (29) had high enrichment between ASB and DSB (Fig. 10a, Additional file 21: Table S12). This suggested that the differences in transcription or translation are the fundamental reason for the difference between ASB and DSB. As for RLB/DSB (Fig. 10b, Additional file 22: Table S13), the photosynthesis (13) pathway had the highest enrichment of DEPs. This is an additional proof for the fact that the main RLB function is photosynthesis for the next clonal generation of ramets. In addition, two highly enriched pathways (DNA replication and spliceosome) were still present indicating that both genetic and transcriptional differences exist between RLB and DSB. The participation of DEPs in the phenylpropanoid biosynthesis pathway (18), as well as in carbon metabolism related pathways, such as starch and sucrose metabolism (15), amino sugar and nucleotide sugar metabolism (14), and glycolysis/gluconeogenesis (11), showed high enrichment between the groups RLB and ASB (Fig. 10c, Additional file 23: Table S14). This indicated that phenylpropaniod biosynthesis caused differences differentiation between RLBs and ASBs in the second node of strawberry stolon, especially with respect to the formation of vessels during the ASB developmental processes, as discussed below.

#### Protein-protein interaction (PPI) analysis

**Fig. 11:**
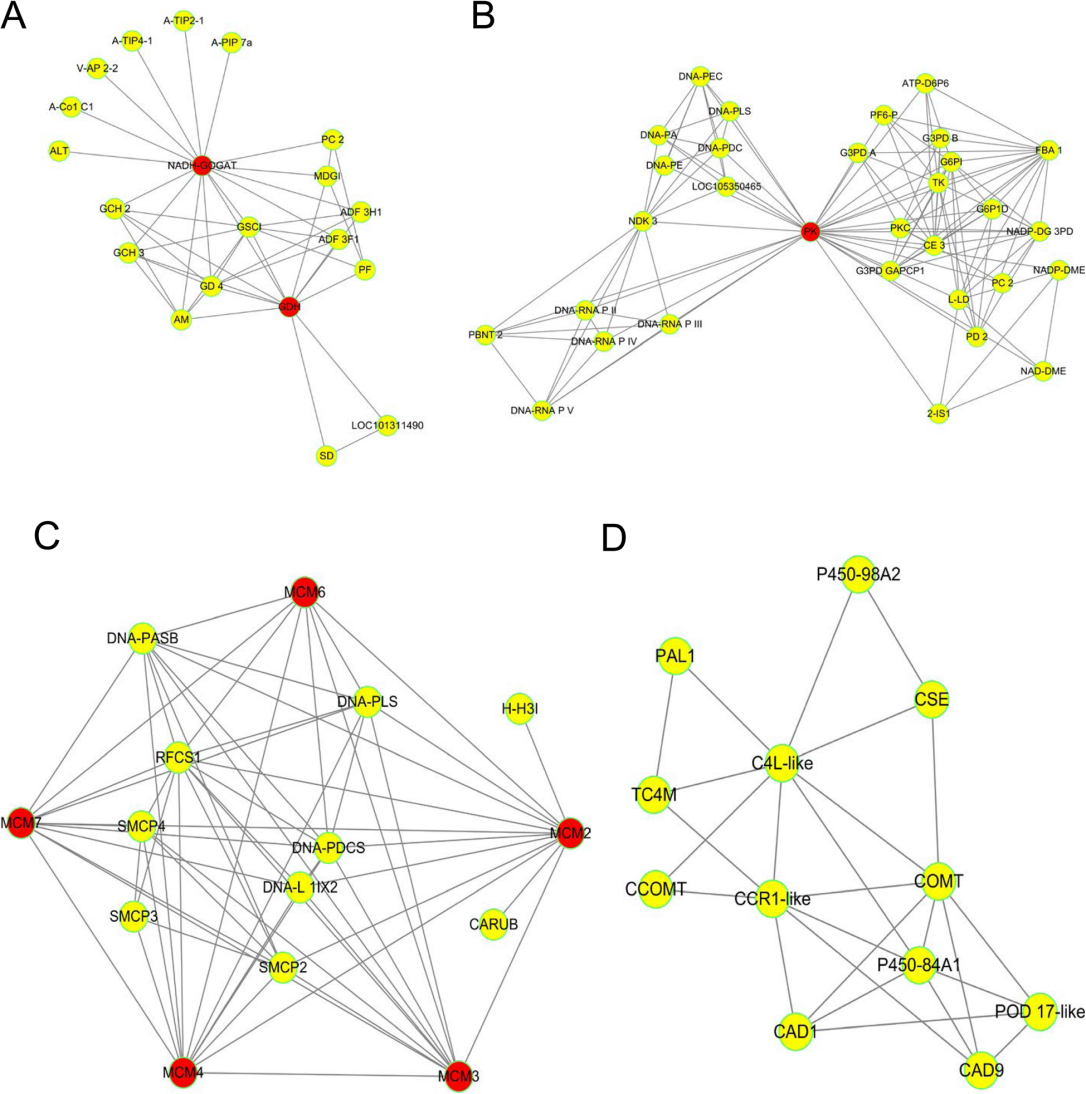
Protein-protein interaction (PPI) of the core proteins NADH-GOGAT, GDH (**a**), and PK (**b**), MCM 2-4 and 6-7 (**c**), as well as the proteins involved in phenylpropanoid biosynthesis pathway (**d**)

The PPI database and relevant literature were used to identify the interactions of the identified proteins or DEPs, as well as of other proteins that interacted directly with them. This PPI network (Additional file 24: Table S15), could providing us a comprehensive information from various points of view, which couldn’t be instated of only a single protein analysis, then the effective protein could be found efficiently (Fig. 11). According to the analysis, 20 high-connectivity degree DEPs, with a degree value of more than 30, were identified between the groups ASB and DSB (Table 2). Six and 11 DEPs, with connectivity degrees higher than 10, were identified from the RLB/DSB and RLB/ASB groups, respectively (Table 2). The results obtained were highly consistent with those obtained using KEGG, indicating that the difference between ASB and DSB was mainly due to the differences at the transcriptional level, while the difference between RLB and DSB was mainly due to the differences at the genetic level. For further validating direct protein-protein interactions, we selected four typical DEPs, namely NADH-GOGAT & GDH, PK, MCM 2–4, and 6–7, as the PPI core (Fig. 11a–c). In additional, we drew the PPIs in the phenylpropanoid biosynthesis pathway (Fig. 11d) to further clarify the key protein-protein interactions.

**Table 2:** DEPs with high connectivity degree in PPI analysis between groups

Group	Protein accession	Degree	Description	Unique peptide	Fold change
ASB/DSB	XP_004304484.1	42	splicing factor 3A subunit 2 isoform X1	5	1.29
	FANhyb_rscf00000157.1.g00010.1	37	60S ribosomal protein L7a-2	6	1.24
	FANhyb_rscf00000522.1.g00007.1	37	small nuclear ribonucleoprotein-associated protein B	3	1.31
	XP_004290666.1	36	small nuclear ribonucleoprotein Sm D1-like	4	1.28
	XP_004303828.1	35	60S ribosomal protein L7-2	2	1.53
	XP_004287907.1	34	U1 small nuclear ribonucleoprotein 70 kDa	10	1.36
	XP_004308170.1	33	40S ribosomal protein S20-2	6	1.28
	XP_004299660.1	33	40S ribosomal protein S6	8	1.25
	FANhyb_rscf00005750.1.g00001.1	33	30S ribosomal protein S17, chloroplastic-like	2	1.27
	XP_004307020.1	33	pyruvate kinase, cytosolic isozyme-like	16	0.83
	XP_004304484.1	42	splicing factor 3A subunit 2 isoform X1	5	1.29
	XP_004297699.1	33	pyruvate kinase, cytosolic isozyme-like	18	0.82
	FANhyb_icon14422311_s.1.g00001.1	32	60S ribosomal protein L27a-3	2	1.28
	XP_004296676.1	32	60S ribosomal protein L27a-3	2	1.24
	XP_004306930.1	32	40S ribosomal protein S13	1	1.62
	FANhyb_rscf00000738.1.g00007.1	32	serine/arginine-rich splicing factor RS31-like isoform X1	7	1.50
	XP_004306844.1	32	serine/arginine-rich splicing factor RS41-like isoform X1	5	1.50
	XP_011470401.1	32	serine/arginine-rich splicing factor RS41 isoform X1	5	1.29
	XP_004300041.1	31	uncharacterized RNA-binding protein C1827.05c	3	1.26
	XP_004299114.1	31	SART-1 family protein DOT2	12	1.26
	XP_004297699.1	33	pyruvate kinase, cytosolic isozyme-like	18	0.82
RLB/DSB	FANhyb_icon00002169_a.1.g00001.1	19	LOW QUALITY PROTEIN: replication factor C subunit 1	1	1.37
	XP_011458268.1	12	DNA replication licensing factor MCM4	18	1.22
	XP_004299199.1	12	protein LIGHT-DEPENDENT SHORT HYPOCOTYLS 10-like	1	3.24
	FANhyb_icon00004233_a.1.g00001.1	12	structural maintenance of chromosomes protein 2-1-like	1	1.46
	XP_011460181.1	11	DNA replication licensing factor MCM3	2	1.26
	XP_004300454.1	11	DNA replication licensing factor MCM7	18	1.27
RLB/ASB	FANhyb_rscf00000086.1.g00005.1	16	glutamate synthase 1 [NADH], chloroplastic isoform X1	20	0.74
	XP_004299600.1	15	acetyl-CoA carboxylase 1-like isoform X1	29	0.65
	XP_004297699.1	14	pyruvate kinase, cytosolic isozyme-like	14	1.23
	FANhyb_icon00042639_a.1.g00001.1	12	hypothetical protein B456_008G262000	1	0.75
	FANhyb_rscf00005750.1.g00001.1	12	30S ribosomal protein S17, chloroplastic-like	2	0.77
	XP_004308170.1	12	40S ribosomal protein S20-2	6	0.81
	FANhyb_rscf00001394.1.g00002.1	12	glutamate dehydrogenase 1	5	2.05
	XP_004287377.1	12	probable histone H2A variant 3	2	0.54
	XP_004309949.1	12	4-coumarate-CoA ligase 2-like	4	0.50
	FANhyb_rscf00001010.1.g00003.1	11	40S ribosomal protein S24-1-like	1	0.80
	FANhyb_rscf00000697.1.g00007.1	11	40S ribosomal protein S24-1	1	0.69

### Parallel reaction monitoring (PRM) verification

**Fig. 12:**
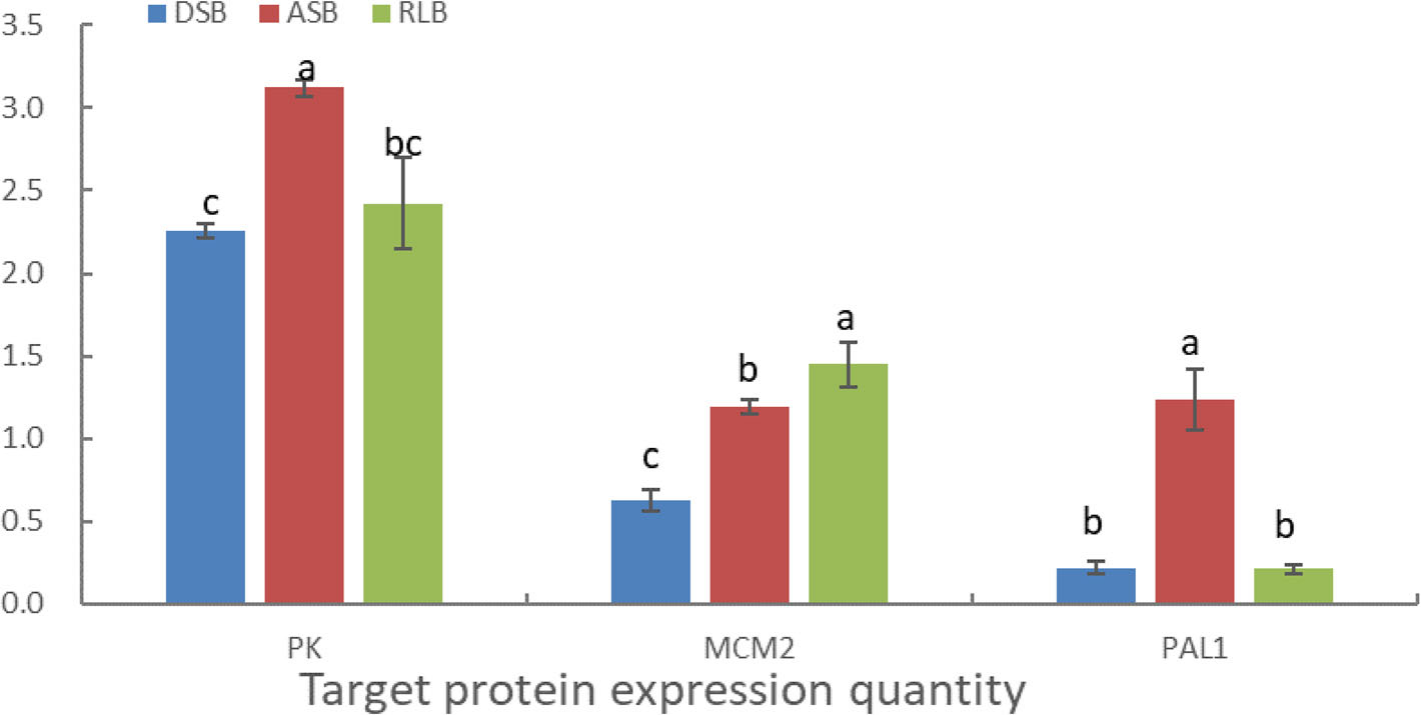
PRM verification of the expression quantities of target proteins PK, MCM2, and PAL1. DSB: dormancy shoot bud; ASB: activity shoot bud; RLB: ramet leaf bud; PK: pyruvate kinase; MCM2: minichromosome maintenance protein 2; PAL1: phenylalanine ammonia-lyase 1. Data are means and standard errors of three groups of each type of bud, and three experimental replicates. Different letters in the same index indicate a significant difference among buds, separately (P<0.05). Bars represent the standard deviation (n=3)

To further verify the results of MS, three DEPs (Pyruvate Kinase (PK), MCM2, and PAL1) were selected for PRM analysis (Fig. 12). The screening criteria were formulated based on the following principles: 1) potential biological functions proteins and their peptide fragments should be greater than 1 (LC-MS/MS identified); and 2) proteins were specifically expressed in one group of buds when compared with the other two groups of buds and have not been reported yet. The results indicated that of the three target proteins, the expression quantities of PK and PAL1 in the ASB group was markedly up-regulated compared with that in the DSB and RLB groups; whereas the expression quantity of MCM2 in the RLB group was significantly up-regulated compared with that in the DSB and ASB groups; these results verified the accuracy of the TMT method for this study.

## Discussion

### Heterogeneity among the buds of different stolon nodes

The stolon is an asexual reproductive organ of strawberry. It is important to study the development process of the buds at different nodes for strawberry production. The stolon of the octoploid cultivated strawberry (*F*. × *ananassa* Duch.) consists of two nodes—the first node usually remains dormant, and the second node has the ability to form the ramets (Fig. 1). A new stolon is usually originats from the axillary bud inside the first leaf of a ramet, in which this bud is mostly conducive to absorbing water and nutrients, and is, therefore, most likely to develop as a new stolon branch [22]. The colonizing behavior and functional morphology of stolons (Fig. 2) indicate that ramet survival, prior to rooting, is achieved through the plasticity of intra-stolon ramet competition for resources such as water, ions, and photoassimilates [8].

By observing the buds on the nodes, we found that the buds on the first node developed into a certain stage and then ceased to develop at the early phase of the primary stolon elongation, indicating their entry into dormancy (Fig. 2 and Fig. 3). Furthermore, under favorable conditions, the buds of the first node can sprout out as another new stolon branches, but these new stolon branches are usually much smaller and thinner than the primary stolon (Fig. 2b). If the apices of a stolon are removed at the appropriate developmental time point, the first node buds do not only release the dormancy, but can also develop into a ramet [3], indicating that the bud in the first node has a binary function of forming a new stolon branch or an independent ramet simultaneously, given that it is an undifferentiated bud or has the ability to dedifferentiate again. However, under normal conditions, the factors that regulate the ceasing of the development of the first node bud and its entry into dormancy are still unknown. We can elaborate the possible factors based on the former studies on the key regulators of axillary bud growth and dormancy in other species. The shoot branching process usually undergo two steps of that the axillary meristems formation, which located in the leaf axils, and the axillary buds growth [23]. In many plant species, the growth of axillary meristems is inhibited by the primary shoot or primary inflorescence, which we called apical dominance [24]. The plant hormones auxin and cytokinin are thought to play a major role in controlling this process [25]. Auxin inhibits the growth of axillary buds, whereas cytokinin promotes axillary bud outgrowth. It depends on the ratio of auxin/cytokinin rather than the absolute levels of either hormone. In the phenotype, the axillary meristems might initiate a few leaves and then become developmentally arrested or dormant because the terminal bud grow predominantly, and inhibits the axillary buds growth [23]. In case of strawberry, we suggest that the development of the first node bud on a stolon is involved in this type of axillary growth; moreover, we observed that axillary meristems initiate a few trifoliolate bracts, as shown in Fig. 2, followed by the cessation of development and entry into dormancy. Consistent with the previous studies, the arrested development of axillary buds in the first node of strawberry and their stepping into dormancy might be comprehensively caused by environmental factors and a feedback to apical dominance.

We know that the axillary buds, located on the leaf axil in the strawberry primary crown, are sensitive to environmental conditions, and perform the different developmental patterns as that 1) stay in dormancy, or 2) develop into either the prostrate long stolon with 2 nodes, as well as 3) another compassed crown branch [11–13]. In the present study, we mainly focused on a single stolon bud’s development and heterogeneity, so we primarily considering whether the environmental elements also regulate the buds heterogeneity and further affect the apical dominance to determine the fate of first node axillary buds in a single stolon. Based on our initial observations, under short photoperiods and with adequate water and fertilizer supply, some of the first node axillary buds could be released from dormancy and then continually develop into a new stolon branch (Fig. 2b); however, the detailed phenological effects need further systematic study.

In addition to environmental considerations, what on earth is the internal factors trigger these fundamental developmental differences between these two axillary buds, located on the first and the second node, separately (Fig. 3 and Fig. 4). Based on reports related to the causes of strawberry axillary bud dormancy, it seems most likely that apical dominance inhibits the axillary bud development of the first node [10, 14, 26]. Previous researches has demonstrated that apical dominance has an inhibitory function in the development of axillary buds into branch crowns and stolons in strawberry, by removing the apices on the compressed primary crown [10, 14]. It is likely that the further development of basal axillary buds in the compressed primary crown is associated with strong apical dominance. As for an independent strawberry stolon, a prostrate branch of the crown, the axillary bud located on the first node, could be viewed as the basal lateral bud when compared with the active developmental apices in the second node; it, thus, seems likely that the dormancy of basal axillary buds on the first node is due to the apical dominance from the second node. That is, the high auxin accumulation, caused by vigorous development of apical meristems in the second node, inhibits the growth of axillary buds in the first node determine that the first node bud stays in dormant after their formation phase.

Thus, as per our phenotypic observation, the dormancy of the first node bud could be released under suitable conditions and its growth could be resumed to develop into a new stolon branch (Fig. 2b). It is possible that a set of genes or proteins that controls the outgrowth or dormancy of axillary buds acts in different phases of the bud developmental processes. A molecular study into this might provide a basis for understanding the regulation of dormant or outgrowing axillary buds in strawberry stolon nodes.

### Proteomics analysis of different stolon buds

Comparative proteomics is a useful approach for identifying functional proteins in illustrating the developmental regulation mechanism of plants [27–30]. Recently, with the tremendous acquisition of the plant reference genome data, more and more comparative proteomics approaches have been applied for studying bud heterogeneity in crops [17–20]. Previous studies compared the proteomes of the first and second nodes of the strawberry stolons to elucidate the internode differences, and found that the DEPs (38 DEPs in the first node, 52 DEPs in the second node) are mostly related to enzymes necessary for carbohydrate metabolism and photosynthesis, in total nine categories were identified: [metabolism], [energy], [photosynthesis], [transcription, protein synthesis], [protein folding, degradation and assembly], [transport], [stress related], [development], and [some unknown proteins] [16]. Some of these DEP categories are similar to those determined by us and are mainly involved in photosynthesis and the developmental processes, between the first and the second node in strawberry stolon. However, the second node of strawberry stolon contains not only a single type of bud, but two types of buds, RLB and ASB; thus, it is necessary to anatomically separate the buds of the second node into two heterogeneous buds before analyzing them. As we mentioned above, in our study, DSB, ASB and RLB on the same stolon were categorized and separated into three groups based on bud type, before performing the TMT, analysis with three biological repeats being set for each group and each repeat containing 180 buds. Approximately 540 fresh stolons, which were in the same growth phase, were anatomically dissected and their DSBs, ASBs, and RLBs were collected as independent samples for experiments in order to eliminate the effect of differences among the experimental individuals and ensure the reliability of the experimental data. The TMT method is more accurate than the traditional two-dimensional gel electrophoresis method for isolating DEPs and can successfully identify DEPs with low expression levels among groups [21]. Therefore, we used the TMT method to explore the primary causes leading to the differences between DSB and ASB, which existed as the stolon axillary shoot buds, but under quite different physiological conditions. In addition, we determined and compared the factors regulating bud differentiation in RLB and ASB, which are commonly located on the second node of a strawberry stolon. Further investigation was conducted to elucidate the reason underlying the development of an axillary ASB from a newly formed ramet, which mainly originates from an RLB and then further develops into an elongated stolon.

### TMT revealed the heterogeneity of stolon buds in strawberry

The proteomics has an important characteristic when compared with the genomics, that is, proteins have a direct influence on each other [31]. The realization of the function of a protein usually depends on its interaction with other proteins, implying that no protein functions independently [32–34]. Therefore, through the comprehensive analysis and evaluation through GO annotation (BP, MF, CC), KEGG pathway enrichment, and determination of connective degree through PPI, we can predict the core functional DEPs involved in the key metabolic pathways [33, 35].

#### Between ASB and DSB

**Fig. 13:**
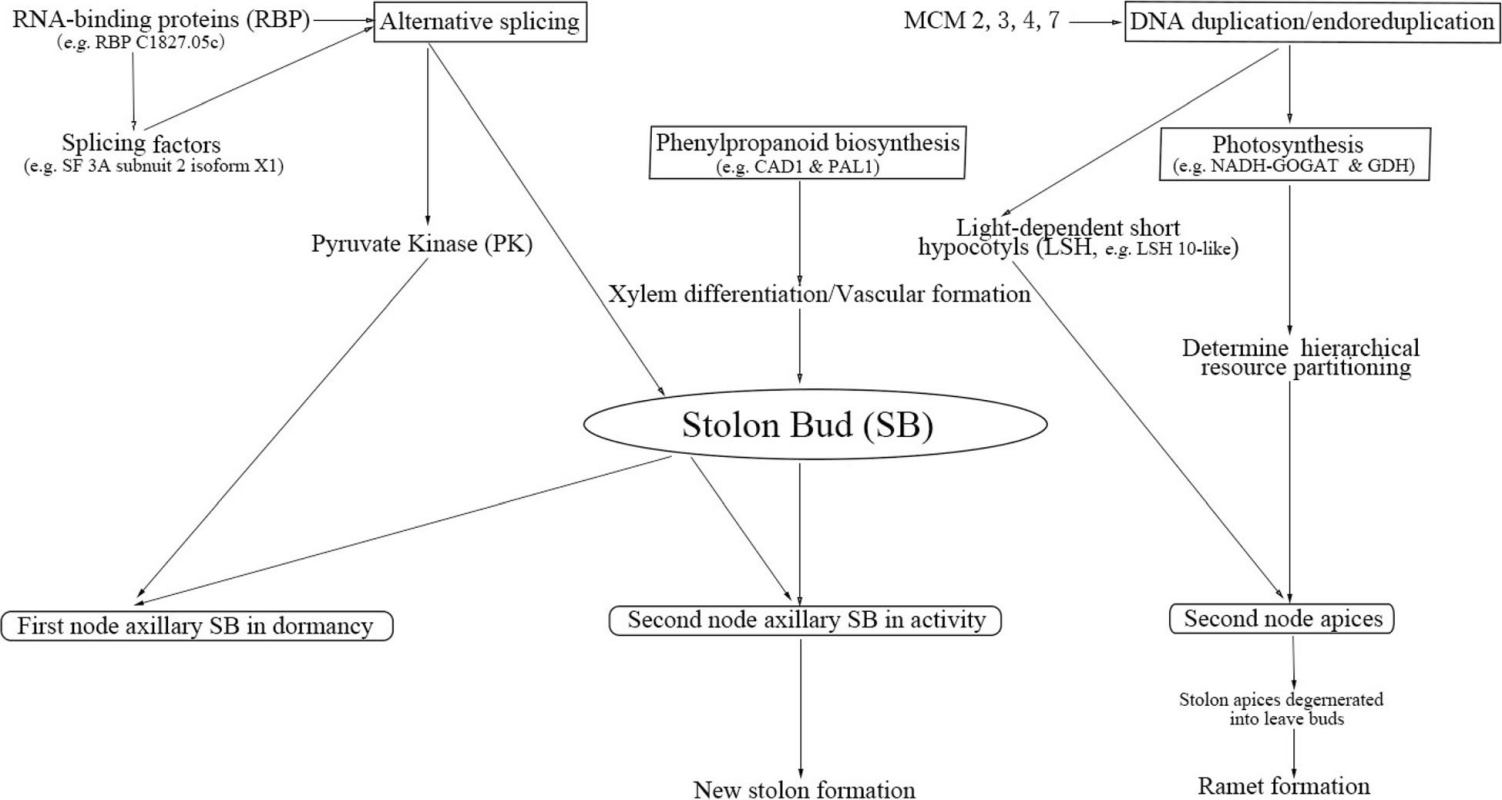
Possible mechanisms for differentially expressed proteins in regulating the heterogeneity of stolon buds in strawberry

According to the comprehensive analysis using GO and KEGG, we found that the differences at the transcriptional level might lead to the differences in their physiological statuses. Upon analysis of PPI data, [splicing factor 3A subunit 2 isoform X1] showed a higher connectivity degree value of 42. As previous studies have reported that alternative splicing has a huge influence on the evolution of complex networks of regulation of gene expression and contributes to the adaptation of plants to their environment, it is thought that alternative splicing can direct strategies for improving plant and crop phenotypes, such as entry into dormancy under stress conditions [36, 37]. Splicing factor (SF), as a positive contributor to the process of alternative splicing, recruits splicing-related proteins and confirms the splicing position and spliceosome assembly; it, thus, participates in the morphological determination of plant organs [38]. SF mainly contained two families of proteins—Ser/Arg-rich (SR) and heterogeneous nuclear ribonucleoprotein particles (hnRNP). In our PPI analysis, of the 19 DEPs, which had high connectivity degree values of more than 30, four (21%) were SRs and six (32%) were hnRNPs. Thus, we suggest that these high connectivity degree SRs and hnRNPs might act as crucial factors in regulating the morphological determination of stolon axillary ASBs and DSBs (Fig. 13).

Similarly, we should also pay attention to the other two DEPs of [pyruvate kinase] (degree 33) and [uncharacterized RNA-binding protein C1827.05c] (degree 31). [Pyruvate kinase] (PK) has been well studied for its ability to modulate bud dormancy or bud break in pomology, and the activity of PK has been found to be lower in dormant buds than in non-dormant buds and to peaked in the green tip stage, just before the start of rapid expansion, and declined thereafter [39–41]. In our study, PK (degree 33) was found to be another highly connective DEP with high expression quantity in ASB, but it was expressed at relatively low levels in DSB and RLB (Fig. 12), suggesting that this pyruvate kinase, cytosolic isozyme functions mainly in ASB rather than in DSB and RLB (Fig. 12). This result was consistent with that of a previous study [39–41], but elucidation of the detailed functional mechanism needs further investigation.

In eukaryotes, RNA-binding proteins (RBPs) play crucial roles in all aspects of post-transcriptional gene regulation. They regulate diverse developmental processes by modulating the expression of specific transcripts. Clearly, they functions by regulating pre-mRNA splicing, polyadenylation, RNA stability, and RNA export, as well as by influencing chromatin modification [36]. The uncharacterized RNA-binding protein C1827.05c (degree31), as a special DEP with a relatively high fold change between groups ASB and DSB, might co-function with the splicing factors and ribosomal proteins (Fig. 13, Additional file 24: Table S15).

#### Between RLB and DSB

The differences between RLB and DSB are mainly due to DEPs involved in photosynthesis (Fig. 9 and Fig. 10); differential expression of these proteins might be caused by the differences at gene duplication level. We identified four DNA replication licensing factors involved in minichromosome maintenance (MCM 2, 3, 4, and 7) from among a total of six high-connectivity degree (>10) DEPs (Additional file 24: Table S15). The DNA replication licensing factor MCM complex, consisting of six subunits, MCM 2–7, is loaded onto the replication origins through loading factors (origin recognition complex [ORC], Cdc6, and Cdt1) and forms an MCM double hexamer that licenses the initiation of DNA replication [42]. The functions of MCM have been mainly studied in medical science, especially in cancer [43]. Here, we supposed that MCM 2, 3, 4, and 7 might upregulate the expression of photosynthetic genes and indirectly regulate photosynthesis substance allocation and transportation by modulating DNA replication or endoreduplication. Our findings were consistent with those of previous studies, which reported that the parenchymal cells that store starch, sugar, and other substances in the fruits or seeds of plants reproduce through DNA replication or endoreduplication [44]. In order to verify the MCM expression mode in strawberry buds, we selected MCM2 as an identified protein and found that it shows a significantly high expression in RLB when compared with DSB and ASB (Fig. 12). Our findings might also partially elucidate the findings of Atkinson et al. (2012), who reported that the hydraulic conductivity and polar auxin transport (PAT) pathways could determine hierarchical resource partitioning and ramet formation in *Fragaria* stolons (Fig. 13) [9].

The DEP with the highest fold change in expression between RLB and DSB was LIGHT-DEPENDENT SHORT HYPOCOTYLS (LSH) 10-like, with a fold change of 3.24 (Table 1, Additional file 24: Table S15). LSH is an important functional regulator of the modulation of the plant shoot initiation process and could be used as a shoot marker for presaging the sites of shoot formation [45, 46]. LSH can be early expressed at the very early stage during zygotic embryogenesis in *Arabidopsis* [45]. As for strawberry, no studies have been conducted on short crown formation. Previous studies showed that differentiated organs can be converted to the other type of organs by various methods; for example, incubation in cytokinin-rich shoot induction medium converts premature roots into shoots, particularly in regions where the cytokinin receptor genes are up-regulated [47]. The flower-meristem-identity gene *LEAFY* is sufficient to determine the floral fate in lateral shoot meristems of both *Arabidopsis* and the heterologous species aspen, with the consequence that flower development is induced precociously [48]. In our study, the LSH 10-like protein was uniquely identified from among a total of 7271 identified proteins by using high-throughput proteomics analysis-based comparison between RLB and DSB. We speculate that the LSH 10-like protein is involved in the degeneration of stolon apical meristem into RLBs (Fig. 13). Further studies are required to investigate how LSH 10-like regulates the formation of a shorted crown of ramet, how ASB could be initiated from the position of a leaf axil in a newly formed ramet, and why the new-born secondary stolon (ASB formed) keeps continuously running along ground, instead of growing upward.

#### Between RLB and ASB

The differences between RLB and ASB in the second node are partially weak compared to those between RLB and DSB. For example, glutamate synthase 1 [NADH], chloroplastic isoform X1 (connectivity degree 15, fold change 0.74, shortened as NADH-GOGAT 1) and glutamate dehydrogenase 1 (connectivity degree 12, fold change 2.05, shortened as GDH 1). NADH-GOGAT and GDH are important enzymes that participate in nitrogen metabolism by synthesizing glutamate [49]. The catalytic function of GDH was direct and more energy-efficient when compared to that of GOGAT [50, 51], and GDH mainly exists in non-photosynthetic tissues of plant, such as root and early development cotyledons [52]. The fold change of NADH-GOGAT was always higher than that of GDH between RLB and ASB.

Another group of special-feature DEPs could be found after a comprehensive comparison between RLBs and ASBs; they are the proteins involved in phenylpropanoid biosynthesis (Ko00940). According to previous reports, the multiple roles of phenylpropanoid biosynthesis in plant development are mainly focused on providing anthocyanins for pigmentation; flavonoids, such as flavones, for protection against UV photodamage; various flavonoid and isoflavonoid inducers of *Rhizobium* nodulation genes; polymeric lignin for structural support; and assorted antimicrobial phytoalexins [53]. In particular, it plays an important role in the differentiation and development of lignin [54]. After comprehensively analyzing the KEGG pathways and PPIs, as well as comparing fold change values among different groups, 18 DEPs highly related to the phenylpropanoid biosynthesis were selected out for further analysis (Fig. 11, Additional file 25: Table S16). Among them, 15 DEPs were up-regulated in the group of ASB/DSB group and down-regulated in the RLB/ASB group simultaneously, and only 3 DEPs showed an opposite trend. In addition, almost all DEPs in the RLB/DSB group showed no significant difference in expression (fold change 0.9–1.2). This means that most DEPs with a phenylpropanoid biosynthesis function were involved in the formation process of ASB (Fig. 13). Cinnamyl alcohol dehydrogenase 1 (CAD1) and phenylalanine ammonia-lyase 1 (PAL1) are only differentially expressed in the RLB/ASB phenylpropanoid biosynthesis pathway. It has been reported that CAD1 and PAL1 are closely related to lignin synthesis [55, 56]. In addition, by determining the expression quantity of PAL1, we further confirmed that the target protein PAL1 was highly expressed in ASB but showed low-level expression in DSB and RLB (Fig. 12). Based on this, we suggest that both CAD1 and PAL1 might play important roles in the axillary bud specialization of a new ramet leaf into ASB. We also speculated that they might play important roles in xylem differentiation or vascular formation when ASB develops into a new stolon (Fig. 13).

## Conclusions

By combining the anatomical observation with the phenotypic observation and using proteomic networks with different types of strawberry stolon buds, we identified the definite dormancy phase of DSB and compared to the developmental differences among DSB, ASB, and RLB. We also identified numerous protein signatures that translated to biological pathways and gene networks that might underlie the real reason of heterogeneity among different stolon buds in strawberry. The possible mechanisms for differentially expressed proteins in regulating the heterogeneity of stolon buds in strawberry were described (Fig. 13). The current study provides further information for understanding the heterogeneity of stolon buds in strawberry, as well as other fruit trees.

## Methods

### Experimental design and statistical rationale

For tandem mass tags (TMT) labeling, each of the three TMT sets included three pooled control samples (*n* = 60 strawberry stolon buds in each sample) for across TMT-plex normalizations, as shown in Fig. 1 (details below). The samples were randomized using an Excel function. The statistical tests used for each experiment are described within each section.

### Plant material

The Ning Yu cultivar of cultivated strawberry (*F.* × *ananassa* Duch.) was grown in the plastic tunnel of the Plant Science Foundation of the Jiangsu Academy of Agricultural Sciences (32.22 °N, 118.52 °E), Nanjing, China, in April 2017. Buds of three physiological statuses of buds in the first node (dormancy shoot bud) and the second node (including activity shoot bud and stolon apices) of a strawberry stolon were selected as materials (Fig. 1). The collected strawberry stolons had a length and diameter of 10 cm and 3 mm, respectively. Three technical replicates were set for each type of bud, with each replicate containing approximately 200 mg, which was collected from 60 buds. More than 540 fresh uniform stolons were collected to obtain these three types of stolon buds as TMT samples. All samples collected were the youngest stolon buds (Figs. 1a–c); they were immediately frozen in liquid nitrogen, and, then, stored at − 80 °C for protein extraction. Triplicates were set for all three types of buds for proteomics analyses.

### Phenotypic observation

In order to observe the morphological differences between the buds of the first and second nodes of the strawberry stolon, the bract of the young buds on the nodes were peeled off using anatomical needles under a stereo microscope (Nikon SMZ 1500). For further observation of the morphological differences among different types of stolon buds located on the first and second nodes, scanning electron microscopy (SEM) observation was used. For SEM observation, different types of stolon bud samples were dehydrated and fixed in 2.5% glutaraldehyde solution. In order to observe the detailed developmental morphological characteristics of the first and second node buds of the strawberry stolon, the stolon was sampled continuously from the beginning (2 cm stolon length and 2 mm diameter) of origination until the second node of the stolon formed an adventitious root primordium (30 cm length and 4 mm diameter). Approximately 20 different developmental phases of strawberry stolon were observed. The stereomicroscopic and SEM observations were all repeated 10 times for the analysis of each developmental phase analysis. Finally, the most characteristic developmental phase of each stolon bud type was determined and was sampled to be used for the proteomics analysis.

### Protein extraction

Based on the anatomical and morphological changes of strawberry stolon buds (Figs. 2 and 5), three types of stolon buds, which were in the most characteristically developed phase, were chosen for quantitative proteomics analysis. Proteins were extracted from young buds using the tricarboxylic acid (TCA)/acetone precipitation and SDT cracking method according to the published protocol [57]. Briefly, approximately 200 mg stolon buds were grounded into a fine powder with liquid nitrogen and homogenized with five volumes of TCA/acetone (1:9). The sample was mixed with whirlpool and precipitated at − 20 °C for more than 4 h and, then, centrifuged at 6000×*g* for 40 min at 4 °C; the supernatant was discarded. The precipitated sample was washed thrice with cold acetone and then dried in ventilator. Subsequently, 20 to 30 mg of the obtained dried powder was weighed and mixed with 30 volumes (m/v) of SDT cracking solution. The precipitate suspension was vortexed, kept in a boiling water bath for 5 min and then subjected to ultrasonic crushing (80 w, 10 s, 15 s intermittent, 10 cycles), incubation in a boiling water bath for 15 min, and centrifugation at 14,000×*g* for 40 min. The protein concentration was determined using a bicinchoninic acid (BCA) protein assay (Bio-Rad, USA) kit according to the manufacturer’s instructions. To confirm protein extraction, one-dimensional SDS-polyacrylamide agarose gel electrophoresis (PAGE) was performed (Additional file 1: Figure S1).

### TMT analysis method

#### Protein digestion and TMT labeling

Protein digestion was performed according to the filter-aided sample preparation FASP procedure described by JR Wisniewski, A Zougman, N Nagaraj and M Mann [58], and the resulting peptide mixture was labeled using the 10-plex TMT reagent according to the manufacturer’s instructions (Thermo Fisher Scientific). Briefly, each sample was dissolved, and 30 μL of the protein solution was mixed with DTT (1,4 dithiothreitol) to the final concentration of 100 mM and incubated in a boiling water bath for 5 min; then, it was cooled to room temperature. Thereafter, 200 μL urea (UA) buffer (8 M Urea, 150 mM TrisHCI, pH 8.0) was added to the solution and mixed evenly. The resulting solution was transferred to a 10-kD ultrafiltration centrifugation tube and centrifuged at 14,000×*g* for 15 min; the supernatant formed was discarded (this step was carried out two times). This was followed by the addition of 100 μL iodoacetamide (IAA) buffer (100 mM IAA in UA), oscillation at 600 rpm for 1 min, refractory reaction at room temperature for 30 min, and centrifugation at 14,000×*g* for 15 min. Subsequently, 100 μL UA buffer was added to the solution and it was centrifuged again at 14,000×*g* for 15 min; this step was repeated two times. Next, 40 μL trypsin buffer [4 μg trypsin in 40 μL 100 mM triethylammonium bicarbonate (TEAB) buffer], was added and the solution was centrifuged at 14,000×*g* for 15 min, and this step was carried out three times. When 40 μL trypsin buffer (4 μg trypsin in 40 μL 100 mM TEAB buffer) was added, oscillated at 600 rpm for 1 min, and placed at 37 °C for 16–18 h. A new collecting tube was replaced and centrifuged at 14,000×*g* for 15 min. Thereafter, 100 mM TEAB buffer was diluted 10 times, and then 40 μL of the diluted TEAB buffer was added and centrifuged again at 14,000×*g* for 15 min. The filtrate was collected, and the peptide segment was quantified (OD280).

For labeling, each TMT reagent was dissolved in 70 μL of ethanol and added to the respective peptide mixture. For each sample, 100 μg of the peptide mixture was labeled using the 10-plex TMT isobaric label reagent (Thermo Fisher Scientific) and then multiplexed and vacuum dried. Samples were labeled as (DSB-1)-126, (DSB-2)-127 N, (DSB-3)-127C, (ASB-1)-128 N, (ASB-2)-128C, (ASB-3)-129 N, (RLB-1)-129C, (RLB-2)-130 N, and (RLB-3)-130C. Then a Pierce high-pH reverse-phase fractionation kit (Thermo Fisher Scientific) was used to make the TMT-labeled and digested samples fractionated into 12 fractions.

### Mass spectrometry

Each fraction was injected for nano-liquid chromatography-tandem mass spectrometry (nano-LC-MS/MS) analysis. The peptide mixture was loaded onto a reverse-phase trap column connected to a C18 reverse-phase analytical column in buffer A (0.1% formic acid) and separated with a linear gradient of buffer B (84% acetonitrile and 0.1% formic acid) at a flow rate of 300 nL/min, controlled by the IntelliFlow technology. The specifications of reverse-phase trap column and C18 reverse-phase analytical column are “Thermo Scientific Acclaim PepMap100, 100 μm × 2 cm, nano Viper, C18” and “Thermo Scientific Easy Column, 10 cm long, 75 μm inner diameter, 3 μm resin”, respectively. The analysis gradient was 1-h long and involved 50 min in 0–50% buffer B, and 5 min holding in 100% buffer B.

The LC-MS/MS analysis was performed on a Q Exactive mass spectrometer (Thermo Fisher Scientific) coupled to an Easy-nLC system (Thermo Fisher Scientific) for 90 min. The mass spectrometer was operated in the positive ion mode. The MS data were acquired using a data-dependent top 10 method, dynamically choosing the most abundant precursor ions from the survey scan (300–1800 m/z) for high-energy collisional dissociation (HCD) fragmentation. The automatic gain control (AGC) target was set to 3E6 and the maximum injection time was set to 10 ms. The duration of dynamic exclusion was 40 s. The survey scans were acquired at a resolution of 70,000 at m/z 200; the resolution of the HCD spectra was set to 35,000 at 20,060; and the isolation width was 2 m/z. The normalized collision energy was 30 eV. The underfill ratio, which specifies the minimum percentage of the target value likely to be reached at the maximum fill time, was defined as 0.1%. The instrument was run with the peptide recognition mode enabled.

### Data analysis

The MS/MS spectra output was obtained as a raw file and were searched using the Mascot engine (Matrix Science, London, UK; version 2.2) embedded into the Proteome Discoverer 1.4 (Proteome Discoverer Version 1.4, Thermo Fisher Scientific Inc. 2012) against the P20180400239_hebing_76764 database (76,764 protein sequences, come from the diploid strawberry (*Fragaria vesca*) genome sequence [4] were combined with the octoploid strawberry (*Fragaria* × *ananassa*) sequencing data [2], which were downloaded from the Genome Database for Rosaceae database https://www.rosaceae.org, and, then, all redundant sequences were removed) and the decoy database. For protein identification, the following options were used. Peptide mass tolerance = ±20 ppm; MS/MS tolerance = 0.1 Da; enzyme = trypsin; max missed cleavage = 2; fixed modification: carbamidomethyl (C), TMT 10 plex (N-term), TMT 10 plex (K); variable modification: oxidation (M); false discovery rate (FDR) ≤ 0.01; protein quantification: the protein ratios are calculated as the median of only unique peptides of the protein; experimental bias: normalizes all peptide ratios by the median protein ratio, and the median protein ratio should be 1 after normalization.

### Bioinformatics analysis

To determine the functional classification and biological properties of the selected differentially expressed proteins (DEPs), the identified protein sequences were mapped using the Gene Ontology (GO) terms. For this analysis, a homology search was performed for all transcript sequences localized in our newly built database P20180400239_hebing_76764.

### Gene ontology and KEGG pathway annotation

The process of GO annotation by Blast2GO can be roughly divided into four steps: (1) sequence alignment (BLAST), (2) GO entry extraction (mapping), (3) GO annotation (annotation), and (4) annotation augmentation (annotation). First, the National Center for Biotechnology Information (NCBI) basic local alignment search tool BLAST+ (ncbi-blast-2.2.28 + −win32.exe) was used to align the target protein set with the appropriate protein sequence database, and the top 10 alignment sequences satisfying the condition E-value less than 1E-3 were retained for subsequent analysis. Second, the mapping process was carried out by using the Blast2GO Command Line to select the relative GO items among the target protein set and qualified items in the first step (Data version: go_201504.obo; download address: http://www.geneontology.org). Third, for GO annotation process, the Blast2GO Command Line took into account the similarity of the target protein sequences and alignment sequences and source reliability of the GO item entries and evaluated the structure of the GO graph. Subsequently, the GO item information was annotated to the target protein, which was selected in the mapping process. Fourth, after annotation, in order to further improve the annotation efficiency, we searched the European Bioinformatics Institute (EBI) database to identify the target proteins by matching conserved motifs using InterProScan [59]. Thereafter, the motif-related functional information was annotated to the target protein, and, then, ANNEX was run to further supplement the annotation information and build the connections among different kinds of GO items for improving the accuracy of annotations. In summary, the GO project described the roles of proteins in three functional categories: biological process (BP), cellular component (CC), and molecular function (MF).

KEGG pathway annotation was used to search and compare genes in the database of KEGG GENES using the KAAS (KEGG Automatic Annotation Server) software [60], followed by preliminary KO classification of target protein sequences. Thereafter, the information on the target proteins involved in the metabolic pathways was automatically obtained according to the KO classification. Finally, the target protein set was comprehensively analyzed using GO ontology or KEGG pathway annotation. To evaluate the protein richness of the GO ontology or KEGG pathways, the Fisher’s exact test applying the hypergeometric test phyper in R was used to compare the distribution of each GO classification or KEGG pathway in the target protein set, followed by the calculation of the significance level.

### Protein clustering

In thermographic clustering analysis, the quantitative information of the target protein set was normalized to a ± 1 interval. Second, the Cluster 3.0 software (https://cluster2.software.informer.com/3.0/) was used to classify the two dimensions of the sample and protein expression simultaneously (distance algorithm: Euclidean; connection mode: Average linkage). Finally, the Java Treeview software was used to generate the hierarchical clustering thermograms.

### Protein-protein interaction network

The gene symbols of the target proteins were firstly obtained from their original databases. Subsequently, the gene symbol information was used to search the database of IntAct (http://www.ebi.ac.uk/intact/main.xhtml) or STRING (http://string-db.org/) to identify direct and indirect interactions among the target proteins according to the experimental evidence. The Cytoscape software (version 3.2.1; http://www.cytoscape.org/release_notes_3_2_1.html) was used to generate the interaction network and analyze it.

### Venn’s diagrams

Venny 2.1.0 (https://bioinfogp.cnb.csic.es/tools/venny/) was used to determine the intersection proteins among the differentially accumulated proteins representing up-regulation or down-regulation.

### Parallel reaction monitoring (PRM) validation

To further check the levels of protein expression determined through TMT analysis, additional quantification was performed through LC-PRM MS analysis according to the protocol of Peterson et al. [61]. The analysis of raw data was realized via Skyline (MacCoss Lab, University of Washington) [62].

### Statistical analysis of data

Data were analyzed using Excel and SPSS by ANOVA followed by Tukey’s significant difference test at *p* ≤ 0.05. All data represent three biological replicates.

## Supplementary information

**Additional file 1: Figure S1.** 1D SDS PAGE for confirming the protein extraction.

**Additional file 2: Figure S2.** Statistical analysis of volcano plot for evaluating the quality of fold change in each group ASB/DSB (A), RLB/DSB (B) and RLB/ASB (C), respectively. Data upon the horizontal and vertical dotted lines, which colored in pink, means the significant changes in the abundance of DEPs with 1.2-fold-change cut-off and *P* value<0.05.

**Additional file 3: Figure S3.** Quality deviation of all identified peptides was mainly within 10 ppm.

**Additional file 4: Figure S4.** Tool of MASCOT in judging each MS2 spectrograms, the median score is 34.06, and more than 86.21% peptides are score higher than 20. Red line means the cumulative curve.

**Additional file 5: Figure S5.** Protein ratio (around 1.0) distribution of groups (A: ASB/DSB; B: RLB/DSB; C: RLB/ASB). FC is short for fold change.

**Additional file 6: Figure S6.** Distribution of the identified protein isoelectric point (main area is concentrated in 5–10, with the mostly PI is 6–7). Red line is the cumulative curve.

**Additional file 7: Figure S7.** The repeatability of quantification data among three biological replicates of each group (A: DSB; B: ASB; C: RLB) according to their quantitative data. Red line means the cumulative curve.

**Additional file 8: Figure S8.** Cluster analysis of differentially expressed proteins. Through horizontal comparison, samples could be classified into three categories, suggesting that the selected DEPs could effectively distinguish samples. Vertical comparison indicated that proteins could be classified into two categories with opposite directional variation, demonstrating the rationality of the selected DEPs. M, N and D correspondingly represent the DSB, ASB and RLB, respectively, (A)—DSB/ASB, (B)—RLB/DSB, (C)—RLB/ASB.

**Additional file 9: Figure S9.** Top 20 DEPs in each terms of GO analysis among ASB/DSB, RLB/DSB, and RLB/ASB.

**Additional file 10: Table S1.** Raw data of identified proteins in TMT quantitation.

**Additional file 11: Table S2**. Raw data of identified peptides in TMT quantitation.

**Additional file 12: Table S3.** Raw data of differentially expressed proteins among DSB-(M), ASB-(N), and RLB-(D).

**Additional file 13: Table S4.** The common DEPs among groups.

**Additional file 14: Table S5.** Unique proteins in all top 10 up- and down-differentially expressed proteins between groups.

**Additional file 15 Table S6.** Co-up regulated DPEs among groups.

**Additional file 16: Table S7.** Co-down regulated DPEs among groups.

**Additional file 17: Table S8.** Total co-up plus co-down regulated DPEs among groups.

**Additional file 18: Table S9.** GO analysis of ASB (N) vs DSB (M).

**Additional file 19: Table S10.** GO analysis of RLB (D) vs DSB (M).

**Additional file 20: Table S11.** GO analysis of RLB (D) vs ASB(N).

**Additional file 21: Table S12.** KEGG analysis of ASB (N) vs DSB (M).

**Additional file 22: Table S13.** KEGG analysis of RLB (D) vs DSB (M).

**Additional file 23: Table S14.** KEGG analysis of RLB (D) vs ASB (N).

**Additional file 24: Table S15. **PPI analysis of connectivity degree among groups.

**Additional file 25: Table S16.** Phenylpropanoid biosynthesis DEPs fold change and PPI analysis.

## Data Availability

All data generated or analyzed in this study are included in this published article and supplementary information files.

## Abbreviations

ASB: Activity shoot bud
DSB: Dormancy shoot bud
RLB: Ramet leave bud
TMT: Tandem mass tags
DEPs: Differentially expressed proteins
SR: Ser/Arg-rich
hnRNP: Heterogeneous nuclear ribonucleoprotein particle
MCM: Minichromosome maintenance protein
CAD1: Cinnamyl alcohol dehydrogenase 1
PAL1: Phenylalanine ammonia-lyase 1
SEM: Scanning electron microscope
TCA: Tricarboxylic acid
BCA: Bicinchoninic acid
PAGE: Polyacrylamide agarose gel electrophoresis
FASP: Filter-aided sample preparation
IAA: Iodoacetamide
TEAB: Triethylammonium bicarbonate
LC-MS/MS: Liquid chromatography-tandem mass spectrometry
HCD: High-energy collisional dissociation
AGC: Automatic gain control
FDR: False discovery rate
GO: Gene Ontology
NCBI: National Center for Biotechnology Information
EBI: European Bioinformatics Institute
BP: Biological process
CC: Cellular component
MF: Molecular function
KEGG: Kyoto Encyclopedia of Genes and Genomes
PRM: Parallel reaction monitoring
*PPI*: Protein-Protein Interaction
PK: Pyruvate Kinase
PAL1: Phenylalanine ammonia-lyase 1
SF: Splicing factor
SR: Ser/Arg-rich
ORC: Origin recognition complex
PAT: Polar auxin transport
LSH: LIGHT-DEPENDENT SHORT HYPOCOTYLS
*LEAFY*: Flower-meristem-identity gene
PI: Isoelectric points
