# Supplementary figures and images for: Phenotypic analysis combined with tandem mass tags (TMT) labeling reveal the heterogeneity of strawberry stolon buds
Source: BMC Plant Biol. 2019 Nov 19;19:505. doi: 10.1186/s12870-019-2096-0 (PMC6862844; doi:10.1186/s12870-019-2096-0)

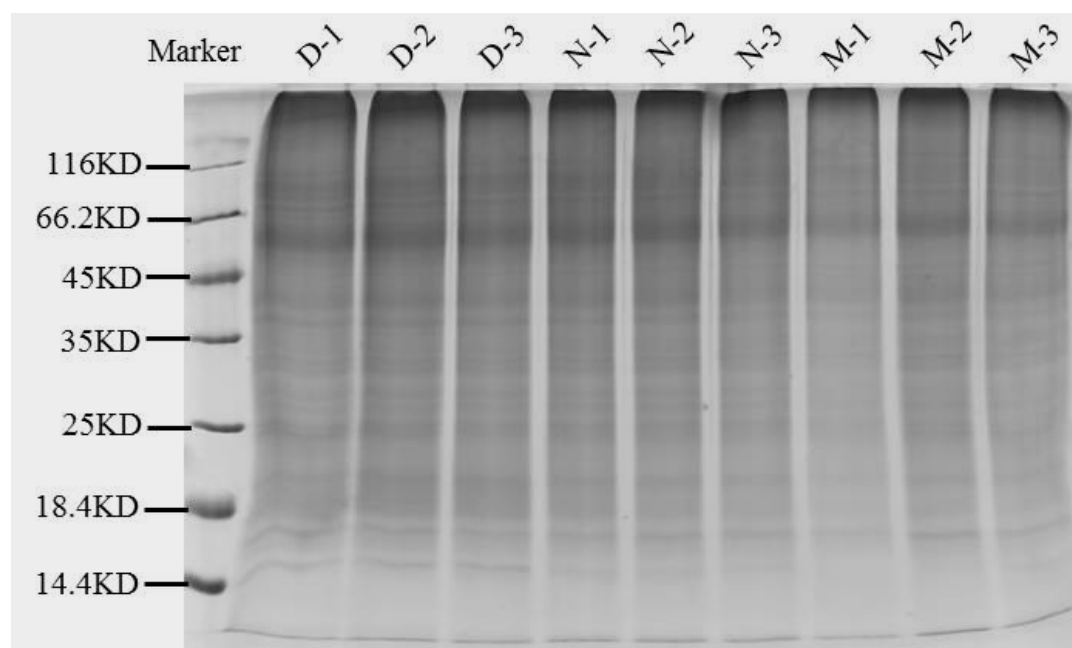

**Supplementary Fig. 1** 1D SDS PAGE for confirming the protein extraction.

Supplement: Supplementary file 1 — Additional file 1: Figure S1. 1D SDS PAGE for confirming the protein extraction. [file 12870_2019_2096_MOESM1_ESM.pdf]

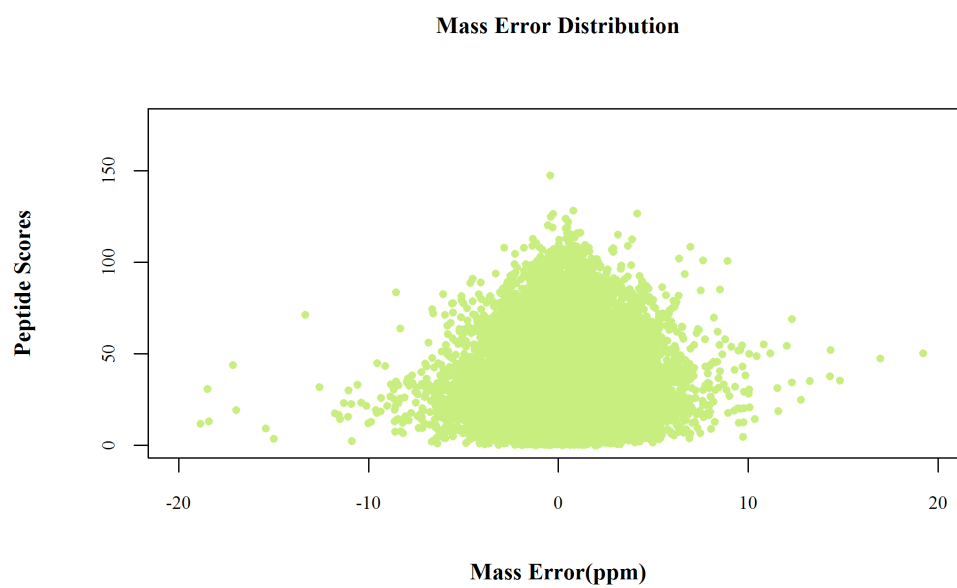

**Supplementary Fig. 3** Quality deviation of all identified peptides was mainly within 10ppm.

Supplement: Supplementary file 3 — Additional file 3: Figure S3. Quality deviation of all identified peptides was mainly within 10 ppm. [file 12870_2019_2096_MOESM3_ESM.pdf]
