# Supplementary material for: Phenotypic analysis combined with tandem mass tags (TMT) labeling reveal the heterogeneity of strawberry stolon buds
Source: BMC Plant Biol. 2019 Nov 19;19:505. doi: 10.1186/s12870-019-2096-0 (PMC6862844; doi:10.1186/s12870-019-2096-0)
Supplement: Supplementary file 2 — Additional file 2: Figure S2. Statistical analysis of volcano plot for evaluating the quality of fold change in each group ASB/DSB (A), RLB/DSB (B) and RLB/ASB (C), respectively. Data upon the horizontal and vertical dotted lines, which colored in pink, means the significant changes in the abundance of DEPs with 1.2-fold-change cut-off and P value<0.05. [file 12870_2019_2096_MOESM2_ESM.pdf]

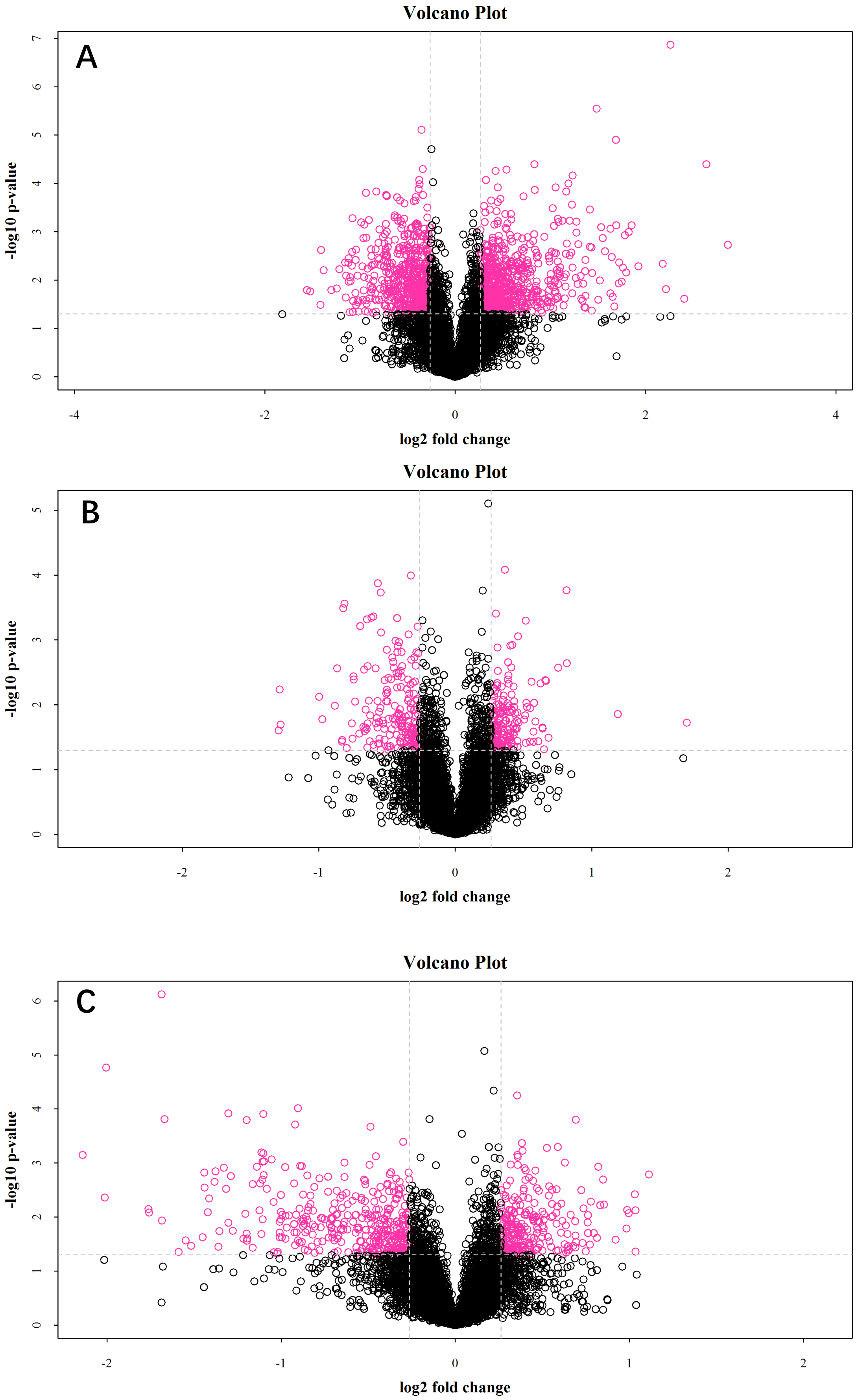
Volcano Plot
A
-log10 p-value
log2 fold change
Volcano Plot
B
-log10 p-value
log2 fold change
Volcano Plot
C
-log10 p-value
log2 fold change

**Supplementary Fig. 2** Statistical analysis of volcano plot for evaluating the quality of fold change in each group ASB/DSB (*A*), RLB/DSB (*B*) and RLB/ASB (*C*), respectively. Data upon the horizontal and vertical dotted lines, which colored in pink, means the significant changes in the abundance of DEPs with 1.2-fold-change cut-off and P value<0.05.
