# Supplementary material for: Phenotypic analysis combined with tandem mass tags (TMT) labeling reveal the heterogeneity of strawberry stolon buds
Source: BMC Plant Biol. 2019 Nov 19;19:505. doi: 10.1186/s12870-019-2096-0 (PMC6862844; doi:10.1186/s12870-019-2096-0)
Supplement: Supplementary file 4 — Additional file 4: Figure S4. Tool of MASCOT in judging each MS2 spectrograms, the median score is 34.06, and more than 86.21% peptides are score higher than 20. Red line means the cumulative curve. [file 12870_2019_2096_MOESM4_ESM.pdf]

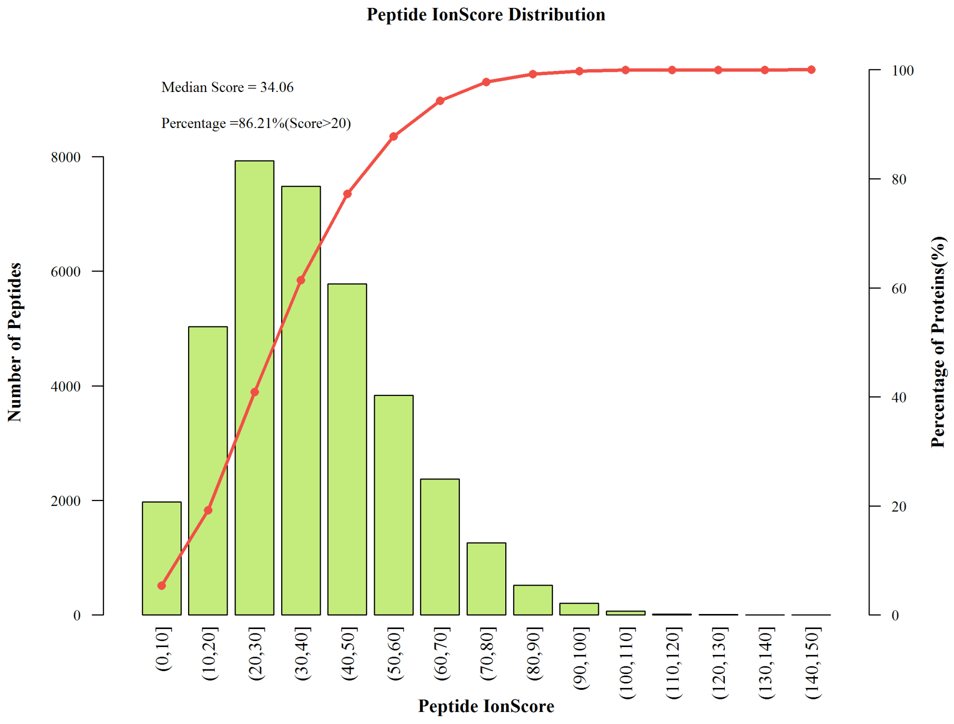

Supplementary Fig. 4 Tool of MASCOT in judging each MS2 spectrograms, the median score is 34.06, and more than 86.21% peptides are score higher than 20. Red line means the cumulative curve.
