# Supplementary material for: Phenotypic analysis combined with tandem mass tags (TMT) labeling reveal the heterogeneity of strawberry stolon buds
Source: BMC Plant Biol. 2019 Nov 19;19:505. doi: 10.1186/s12870-019-2096-0 (PMC6862844; doi:10.1186/s12870-019-2096-0)
Supplement: Supplementary file 5 — Additional file 5: Figure S5. Protein ratio (around 1.0) distribution of groups (A: ASB/DSB; B: RLB/DSB; C: RLB/ASB). FC is short for fold change. [file 12870_2019_2096_MOESM5_ESM.pdf]

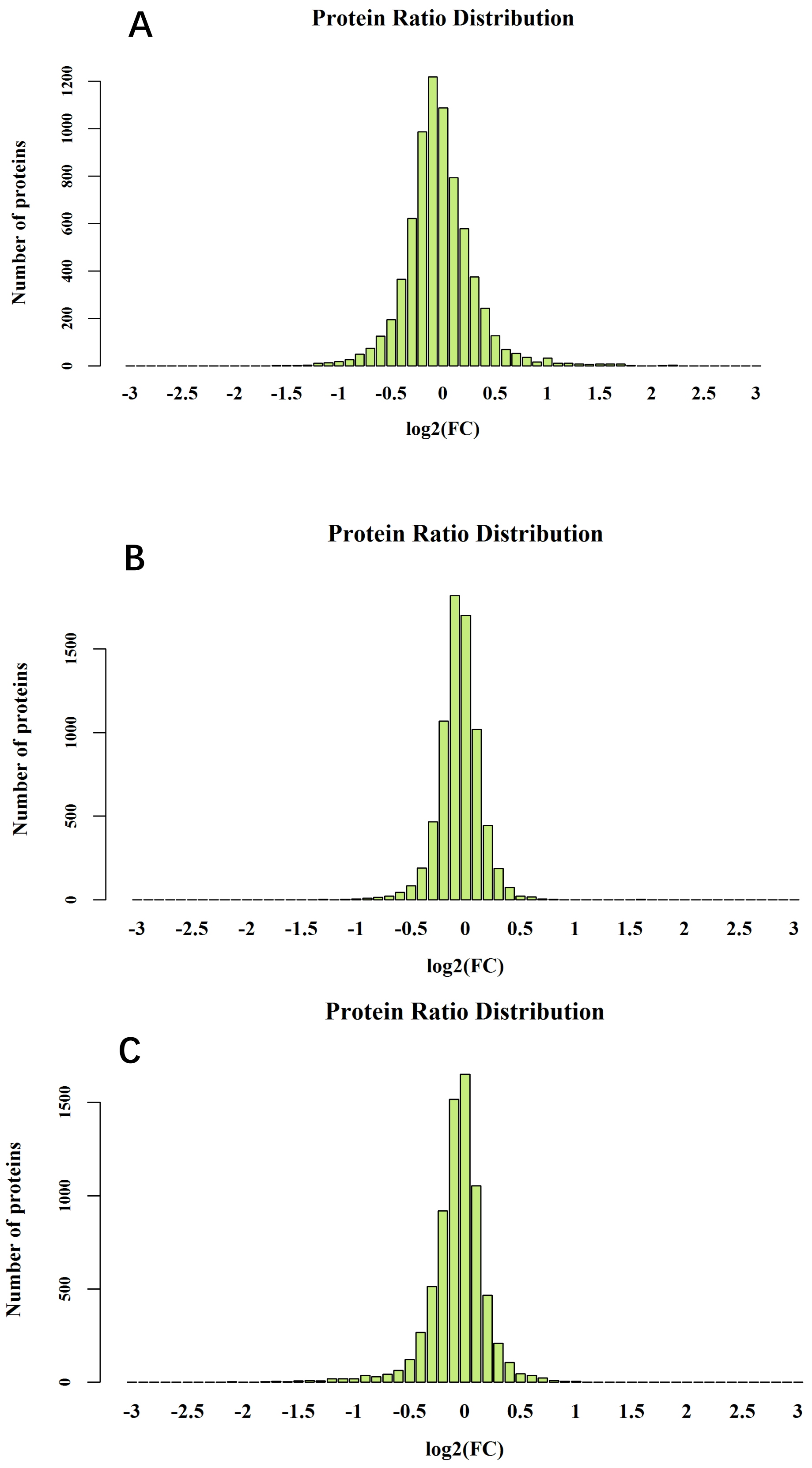


**Supplementary Fig. 5** Protein ratio (around 1.0) distribution of groups

(A: ASB/DSB; B: RLB/DSB; C: RLB/ASB). FC is short for fold change.
