# Supplementary material for: Phenotypic analysis combined with tandem mass tags (TMT) labeling reveal the heterogeneity of strawberry stolon buds
Source: BMC Plant Biol. 2019 Nov 19;19:505. doi: 10.1186/s12870-019-2096-0 (PMC6862844; doi:10.1186/s12870-019-2096-0)
Supplement: Supplementary file 6 — Additional file 6: Figure S6. Distribution of the identified protein isoelectric point (main area is concentrated in 5–10, with the mostly PI is 6–7). Red line is the cumulative curve. [file 12870_2019_2096_MOESM6_ESM.pdf]

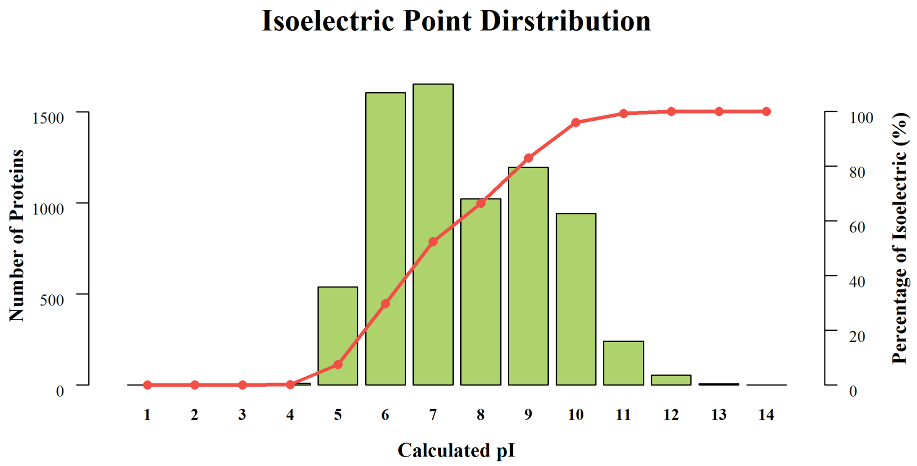


**Supplementary Fig. 6** Distribution of the identified protein isoelectric point (main area is concentrated in 5-10, with the mostly PI is 6-7). Red line is the cumulative curve.
