# Supplementary material for: Phenotypic analysis combined with tandem mass tags (TMT) labeling reveal the heterogeneity of strawberry stolon buds
Source: BMC Plant Biol. 2019 Nov 19;19:505. doi: 10.1186/s12870-019-2096-0 (PMC6862844; doi:10.1186/s12870-019-2096-0)
Supplement: Supplementary file 7 — Additional file 7: Figure S7. The repeatability of quantification data among three biological replicates of each group (A: DSB; B: ASB; C: RLB) according to their quantitative data. Red line means the cumulative curve. [file 12870_2019_2096_MOESM7_ESM.pdf]

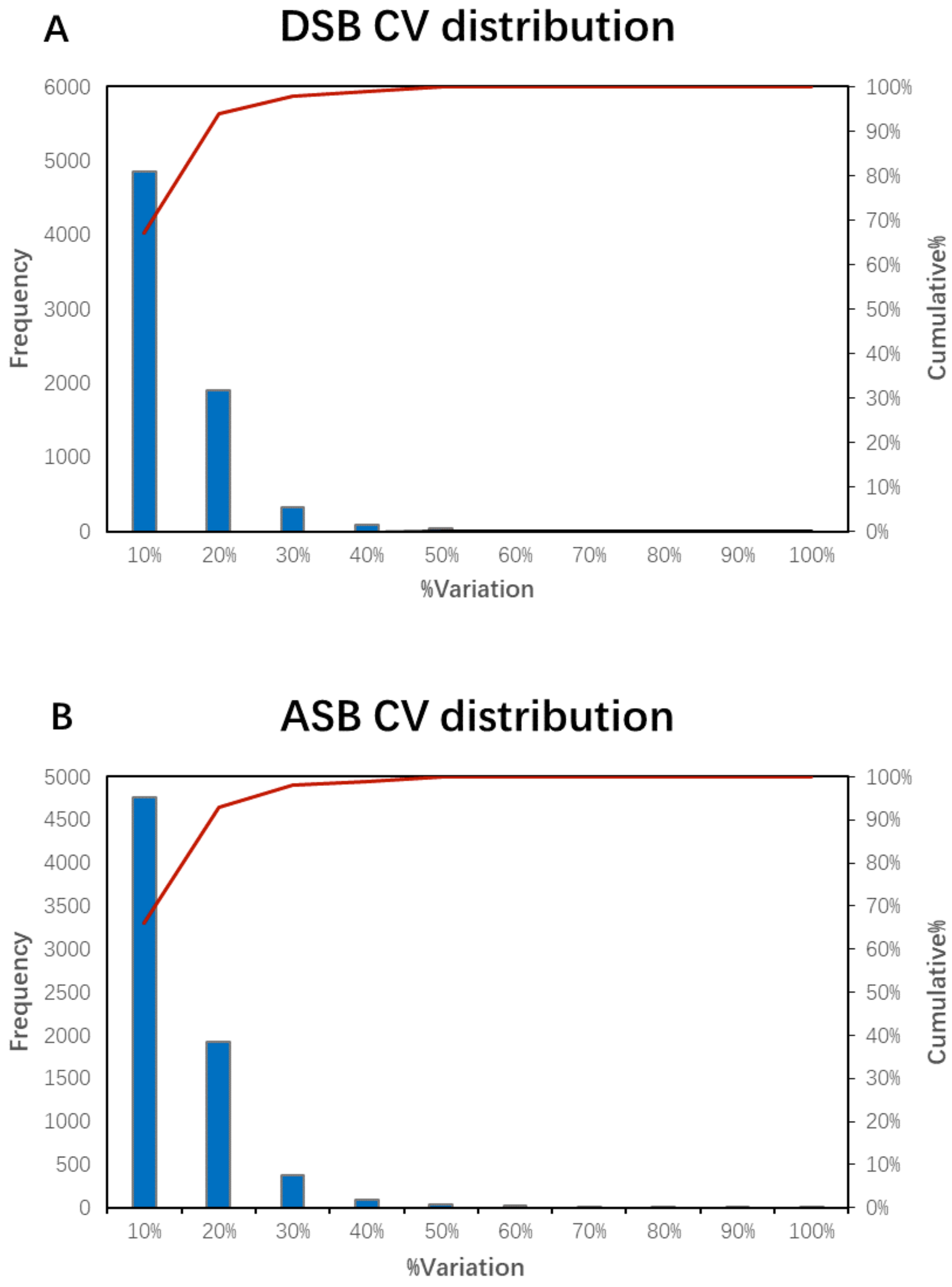
A
DSB CV distribution
Frequency
6000
5000
4000
3000
2000
1000
0
Cumulative%
100%
90%
80%
70%
60%
50%
40%
30%
20%
10%
0%
10%
20%
30%
40%
50%
60%
70%
80%
90%
100%
%Variation
B
ASB CV distribution
Frequency
5000
4500
4000
3500
3000
2500
2000
1500
1000
500
0
Cumulative%
100%
90%
80%
70%
60%
50%
40%
30%
20%
10%
0%
10%
20%
30%
40%
50%
60%
70%
80%
90%
100%
%Variation

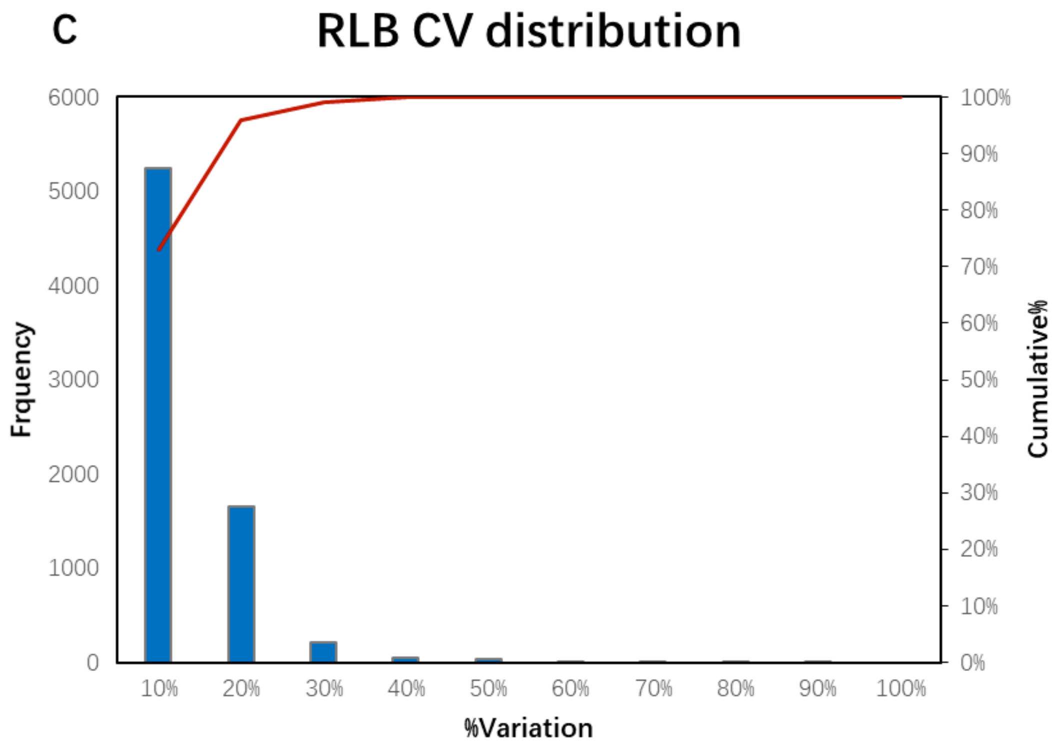

Supplementary Fig. 7 The repeatability of quantification data among three biological replicates of each group (A: DSB; B: ASB; C: RLB) according to their quantitative data. Red line means the cumulative curve.
