# Supplementary material for: Phenotypic analysis combined with tandem mass tags (TMT) labeling reveal the heterogeneity of strawberry stolon buds
Source: BMC Plant Biol. 2019 Nov 19;19:505. doi: 10.1186/s12870-019-2096-0 (PMC6862844; doi:10.1186/s12870-019-2096-0)
Supplement: Supplementary file 8 — Additional file 8: Figure S8. Cluster analysis of differentially expressed proteins. Through horizontal comparison, samples could be classified into three categories, suggesting that the selected DEPs could effectively distinguish samples. Vertical comparison indicated that proteins could be classified into two categories with opposite directional variation, demonstrating the rationality of the selected DEPs. M, N and D correspondingly represent the DSB, ASB and RLB, respectively, (A)—DSB/ASB, (B)—RLB/DSB, (C)—RLB/ASB. [file 12870_2019_2096_MOESM8_ESM.pdf]

A

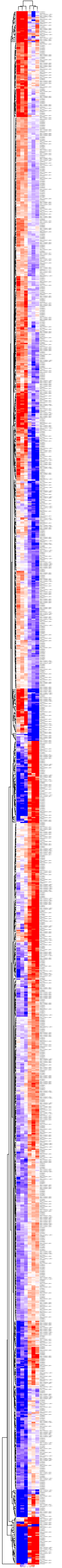

B

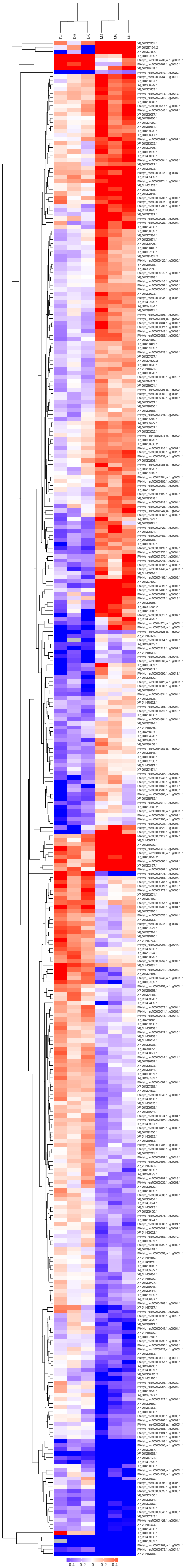

C

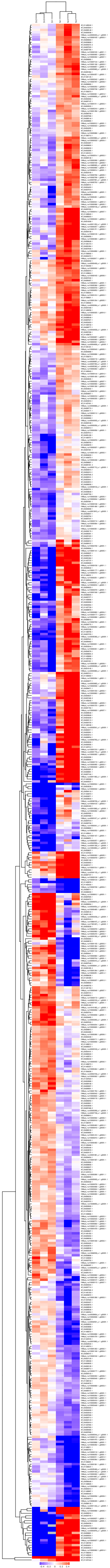

Supplementary Fig. 8 Cluster analysis of differentially expressed proteins. Through horizontal comparison, samples could be classified into three categories, suggesting that the selected DEPs could effectively distinguish samples. Vertical comparison indicated that proteins could be classified into two categories with opposite directional variation, demonstrating the rationality of the selected DEPs. M, N and D correspondingly represent the DSB, ASB and RLB, respectively, (A)—DSB/ASB, (B)—RLB/DSB, (C)—RLB/ASB.
