# Supplementary material for: Phenotypic analysis combined with tandem mass tags (TMT) labeling reveal the heterogeneity of strawberry stolon buds
Source: BMC Plant Biol. 2019 Nov 19;19:505. doi: 10.1186/s12870-019-2096-0 (PMC6862844; doi:10.1186/s12870-019-2096-0)
Supplement: Supplementary file 9 — Additional file 9: Figure S9. Top 20 DEPs in each terms of GO analysis among ASB/DSB, RLB/DSB, and RLB/ASB. [file 12870_2019_2096_MOESM9_ESM.pdf]

A. Go analysis in ASB/DSB

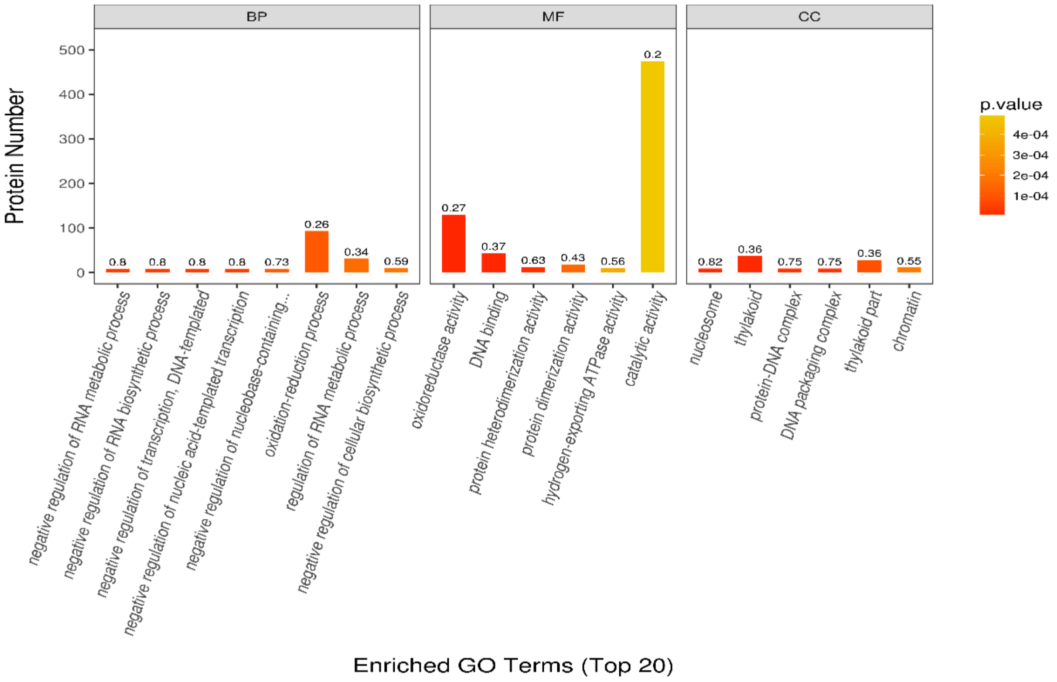

B. Go analysis in RLB/DSB

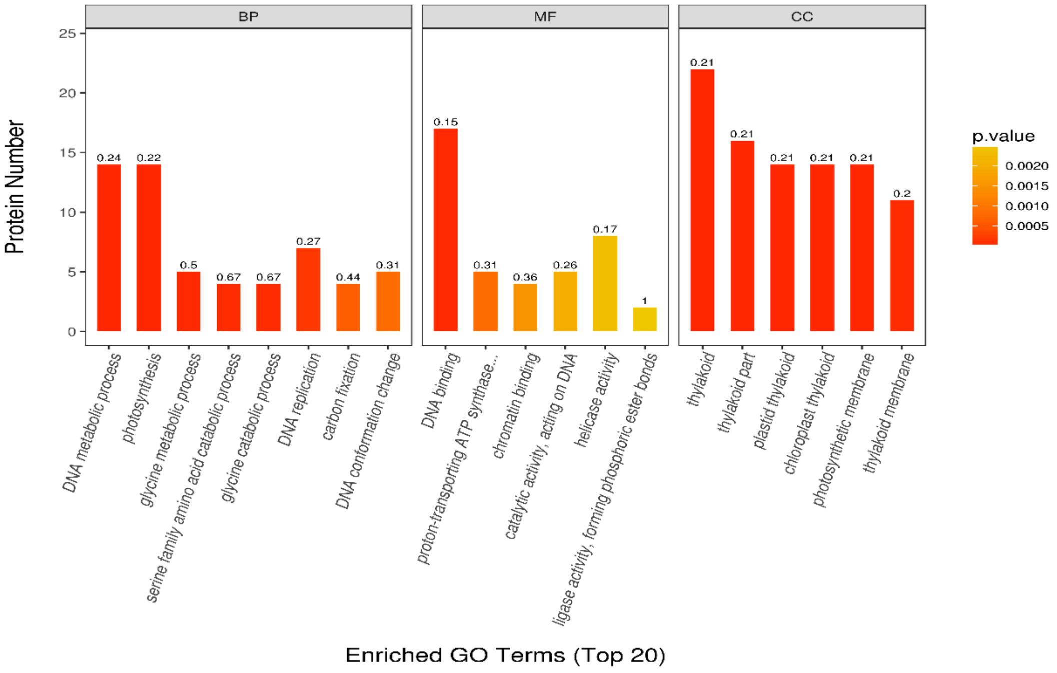

C. Go analysis in RLB/ASB

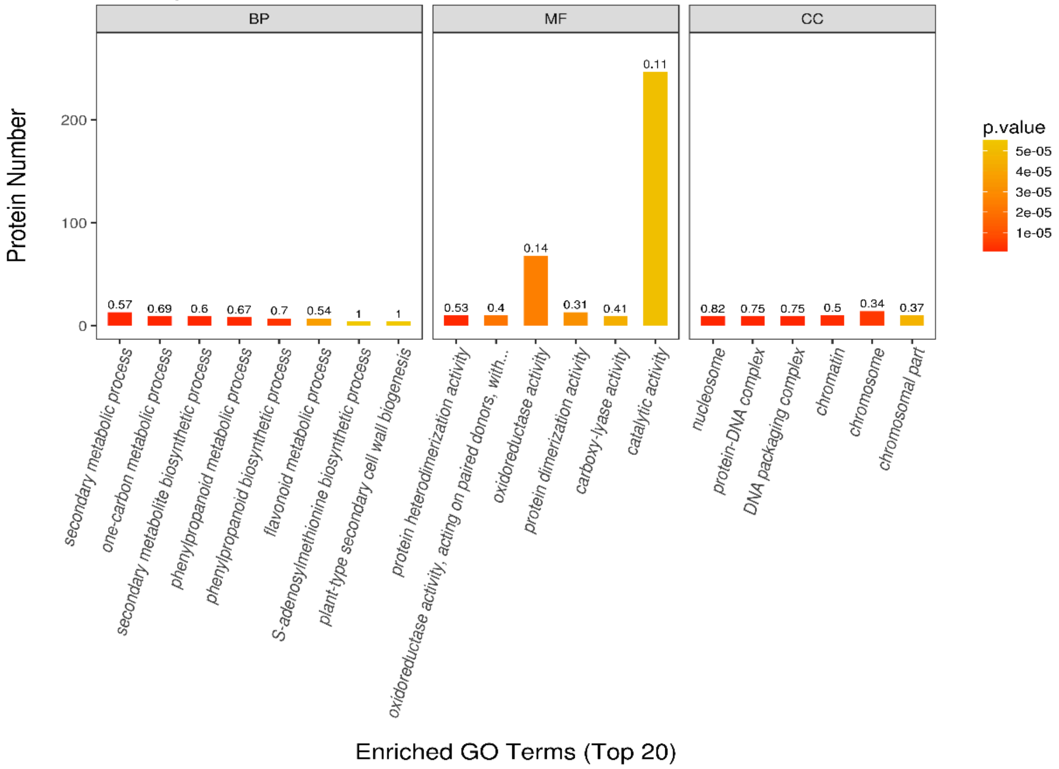

Supplementary Fig. 9 Top 20 DEPs in each terms of GO analysis among ASB/DSB, RLB/DSB, and RLB/ASB.
